# Optimization of Hunhe waterfront parks in Shenyang based on Attention Restoration Theory

**DOI:** 10.3389/fpubh.2025.1695578

**Published:** 2025-11-14

**Authors:** Yifei Wang, Jingmei Zhai, Jian Zhuo

**Affiliations:** 1 School of Architecture and Urban Planning, Tongji University, Shanghai, China; 2 Urban Renewal Planning Research Center, Shanghai Tongji Urban Planning & Design Institute Co., Ltd., Shanghai, China; 3 Yantai Municipal Bureau of Natural Resources and Planning, Yantai, China

**Keywords:** waterfront park, Attention Restoration Theory, restorative environmental factors, multidimensional evaluation model, healthy cities, the Hun River

## Abstract

**Introduction:**

Rapid urbanization and increasing environmental pressures have intensified public health challenges, making the restorative potential of urban blue–green spaces a growing concern. Urban waterfront parks, as critical ecological and social infrastructures, play a significant role in promoting psychological recovery and physiological well-being. This study investigates how the spatial attributes and behavioral patterns of waterfront parks jointly shape restorative mechanisms, and how differentiated spatial optimization strategies can be proposed accordingly.

**Methods:**

Seven representative waterfront parks along the Hun River in Shenyang, China, were examined. Drawing upon Attention Restoration Theory, the study integrates objective spatial indicators and subjective perception data to construct a multi-dimensional evaluation framework. Methods including spatial analysis, questionnaire surveys, and multivariate statistical modeling were employed to identify the underlying mechanisms linking environmental qualities, user behaviors, and health restoration effects.

**Results:**

The findings reveal a multi-level interaction between spatial configuration, environmental perception, and behavioral participation in shaping restorative experiences. Different parks demonstrate distinct restorative orientations—socially interactive, psychologically reflective, and physiologically restorative—corresponding to variations in spatial form and user engagement. These results underscore that health restoration emerges not from single spatial elements, but from the synergistic relationship between environmental structure, sensory stimuli, and behavioral adaptability.

**Discussion:**

The study establishes an integrated theoretical and analytical framework for assessing and enhancing the restorative potential of urban waterfront parks. It offers both conceptual insights and practical guidance for health-oriented spatial planning, contributing to the creation of adaptive, resilient, and human-centered public spaces in high-density urban environments.

## 1 Introduction

Against the backdrop of rapid urbanization and industrialization, Chinese cities have achieved remarkable economic growth and spatial expansion, yet simultaneously face a series of structural challenges (1–3). The extensive development model, characterized by high-intensity construction and excessive resource consumption (4, 5), has led to a range of “urban diseases,” including excessive spatial density, disorderly expansion, and severe traffic congestion (6, 7). As residents continue to live in such highly stressful environments, their physical and mental health is being significantly undermined (8, 9). With the ongoing growth of urban populations and the acceleration of social rhythms, public demand for healthier, more comfortable, and sustainable living environments has become increasingly urgent (10). Consequently, how to achieve proactive health interventions through urban spatial design (11–13) has emerged as a pressing issue in contemporary urban planning.

Within this context, waterfront parks-representing a typical form of urban green and blue infrastructure-have become essential spaces for residents to relieve stress, restore health, and enhance well-being, owing to their unique natural resource endowments and superior environmental quality (14–18). Water landscapes not only exert strong visual appeal (19, 20) but also improve immune function, regulate sleep quality, and promote psychological balance by releasing negative ions and moderating microclimates (21–23). The physiological relaxation and psychological pleasure provided by waterfront environments underscore their irreplaceable role in health-oriented urban spatial systems (24–27). From an international perspective, waterfront areas have gradually shifted from traditional functional shorelines to multifunctional spaces integrating ecological, recreational, and health-promoting values, serving as important drivers for urban revitalization and spatial governance (28, 29).

Since the mid-20th century, international scholarship has increasingly emphasized the multifaceted social and ecological roles of urban waterfronts, advocating for the redevelopment of old docks (30), derelict industrial lands (31), and similar sites into culturally distinctive and ecologically functional waterfront parks, thereby fostering urban vitality regeneration (32). However, most of these international studies were conducted in waterfront spaces with medium to low usage intensity, which differs substantially from the high-density, high-intensity use conditions typical of Chinese cities. In contrast, research on waterfront spaces in China started relatively late. Existing studies largely focus on landscape aesthetics (33) and ecological restoration (34) from a landscape architecture perspective, while urban planning research has paid insufficient attention to health functions of waterfront parks. Moreover, there remains a lack of systematic evaluation frameworks and classification-based optimization strategies in this field. Specifically, the restorative value of waterfront parks characterized by the dual attributes of blue and green environments and high usage intensity remains underexplored. Many Chinese urban parks, particularly in dense cities such as Shenyang, combine ecological, recreational, and social functions within limited space. Therefore, it is also necessary to consider the impact of indicators such as slow-moving network density and facility diversity on the health functions of waterfront spaces.

On the other hand, the theory of restorative environments provides a solid foundation for understanding the mechanisms through which natural environments contribute to health recovery. Represented by Kaplan and Kaplan’s Attention Restoration Theory (ART) (35), international studies have established a relatively mature theoretical system, covering multiple dimensions such as the relationship between natural elements and health benefits (36–38), comparisons of restorative effects between natural and artificial environments (39, 40), and the differentiated health impacts of diverse landscape characteristics (41, 42). In contrast, relevant research in China only began around 2015, focusing primarily on urban comprehensive parks (23), community green spaces (43, 44), and university campuses (45). However, the restorative value of waterfront parks-spaces characterized by the dual attributes of blue and green environments and high usage intensity-remains underexplored. Moreover, most existing studies emphasize therapeutic landscape design (46–48), with relatively few addressing macro-level spatial organization and its coupling with behavioral characteristics, thus lacking systematic and operational analytical frameworks. To address these gaps, this study introduces Attention Restoration Theory into the research of urban waterfront parks from an urban spatial planning perspective, extending its theoretical and practical boundary. By integrating the blue-green attributes of waterfront environments with users’ behavioral patterns, this research constructs a framework that links perceptual environmental characteristics, behavioral activities, and health restoration outcomes, aiming to establish a new analytical paradigm that enhances both the theoretical interpretability and spatial operability of ART in the Chinese urban context.

The Hunhe waterfront in Shenyang serves as a key node at the intersection of the city’s north–south “Golden Corridor” development axis and the Hunhe ecological belt. It not only accommodates ecological, cultural, and recreational functions but also represents a strategic space for driving urban regeneration and transformation. As a high-density city in Northeast China, Shenyang provides a unique context where waterfront parks integrate ecological quality with intensive public use, making it an ideal case to study the restorative potential of blue-green dual-attribute spaces under high usage intensity. In recent years, Shenyang’s continuous investment in the planning and construction of the Hunhe waterfront has expanded its spatial scope from the central urban section to broader city areas, making it an essential platform for restructuring urban form and enhancing living quality. Therefore, taking the Hunhe Riverside Park as the research object, this study explores its spatial performance and optimization strategies from the perspective of restorative health benefits, highlighting both theoretical significance and practical value.

In light of this, this study takes representative waterfront parks along the Hun River in Shenyang as its research object. The main research question centers on how spatial attributes and behavioral patterns of blue-green waterfront parks jointly shape the mechanisms of psychological and physiological restoration, and how differentiated spatial optimization strategies can be developed based on these restorative characteristics. Drawing upon Attention Restoration Theory, the study develops a comprehensive evaluation framework that integrates perceptual environmental attributes with behavioral experience factors, identifies key environmental elements influencing health recovery effects through quantitative analysis, and classifies park types accordingly.

The significance of this research lies in two main aspects. First, at the theoretical level, it offers a new perspective on the role of natural environments in addressing public health issues, fills the research gap regarding the restorative benefits of urban blue spaces, and enriches the theoretical framework of healthy urban spaces. Second, at the practical level, it responds to the ongoing shift in urban governance from “passive treatment” to “proactive intervention,” explores the active role of urban planning in mitigating residents’ mental stress, preventing chronic diseases, and improving lifestyles, and provides implementable strategic suggestions for functional enhancement and spatial optimization of waterfront parks.

## 2 Materials and methods

### 2.1 Study area and research objects

**Figure 1:**
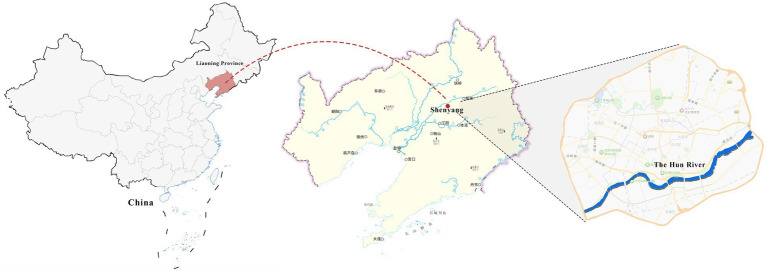
Shenyang (Hunhe) geographical location.

The Hun River, often referred to as the “Mother River” of Shenyang, originates from Gunmaling in the Changbai Mountain range, flows through Fushun and Shenyang, and eventually joins the Liao River. With a total length of approximately 415 km, the section within Shenyang’s administrative boundary extends about 173 km. As a major water system traversing the southern part of Shenyang (Figure 1), the Hun River not only constitutes a critical component of the regional ecological pattern but also plays a pivotal role in shaping the city’s image, supporting waterfront development, and providing public space.

In recent years, Shenyang has advanced the strategic plan of “One River, Two Banks,” designating the Hun River as the north–south development axis of the city and setting the overarching goal of creating a world-class waterfront district. Based on the existing spatial development conditions, the strategy divides the river corridor into several functional segments. Among them, the “Flourishing Nine Kilometers” core section-stretching westward from Shengli Bridge to eastward Changqing Bridge-encompasses key urban functional areas such as Changbai, Wulihe, and the Olympic Sports Center. Situated within the central districts of Shenyang (Heping, Shenhe, and Hunnan), this corridor represents one of the most intensely developed and heavily utilized waterfront zones, with the highest concentration of urban public spaces.

**Figure 2:**
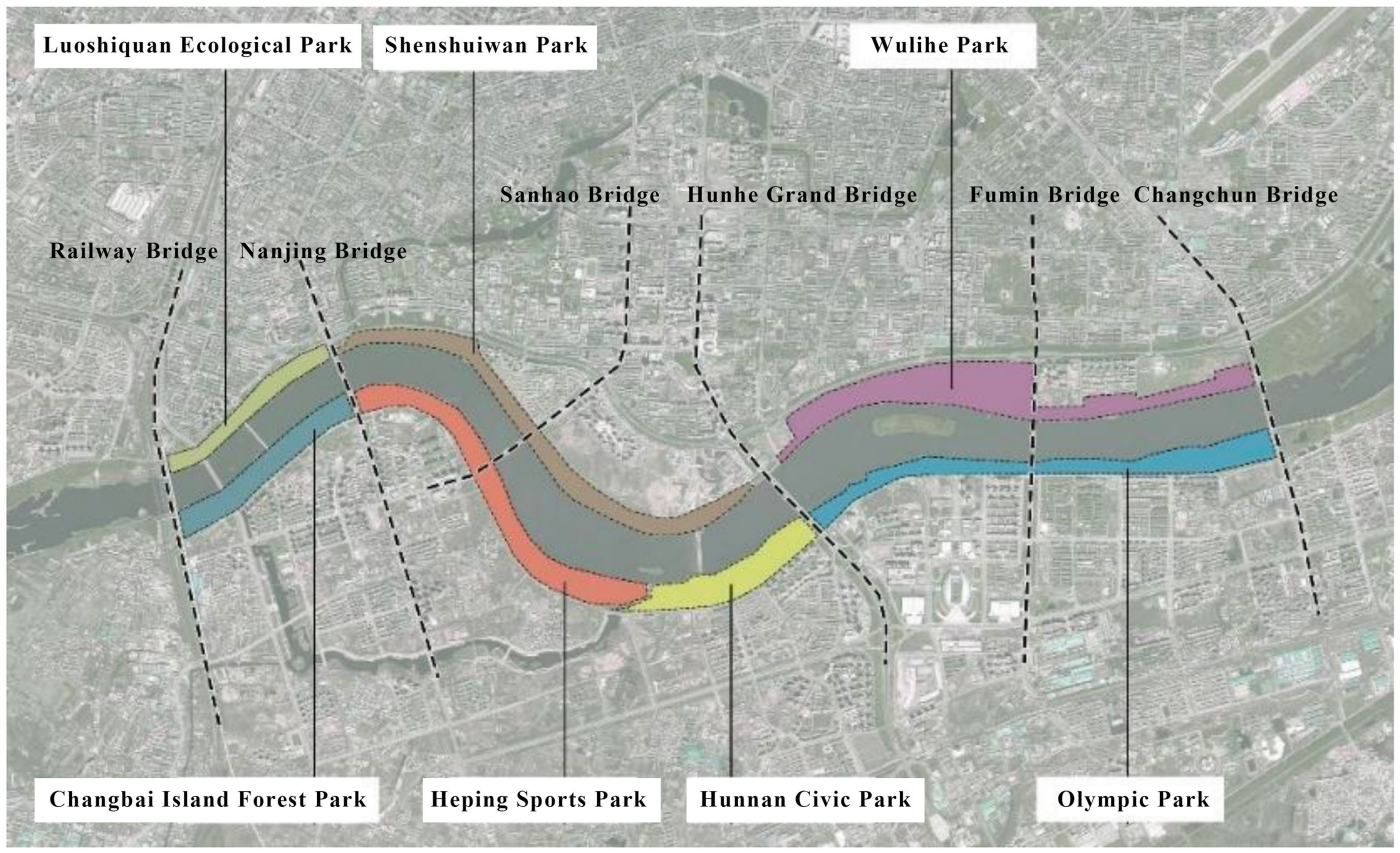
Study area and research objects.

This study focuses on seven representative waterfront parks within the “Flourishing Nine Kilometers” section (Figure 2): Luoshichuan Ecological Park, Shenshuiwan Park, Wulihe Park, Changbai Island Forest Park, Heping Sports Park, Hunnan Citizens’ Park, and the Olympic Park. Extending along a total length of about 13 km, this area is characterized by high functional diversity, intensive human activity, and well-developed park facilities, providing an ideal setting for empirical research on restorative environments. The selected parks differ in terms of geographic location, landscape typology, user groups, and modes of utilization, thereby offering a representative sample base for identifying the key factors influencing health-restorative effects in waterfront spaces and exploring pathways for classification-based optimization.

### 2.2 Data sources

The data employed in this study consist of two main categories: objective data and subjective data. The objective data were obtained through web scraping, remote sensing image interpretation, and on-site field surveys. These datasets included quantitative indicators such as vegetation coverage, non-motorized road network density, revetment types, environmental noise levels, water landscape aesthetics, and the number and quality of recreational and fitness facilities. Together, these indicators provide an objective representation of the natural environment, spatial structure, and facility configuration of the waterfront parks.

The subjective data were collected via a structured questionnaire survey, focusing on public perceptions of psychological and experiential aspects such as spatial safety, visual openness, and color perception. These subjective assessments complement the objective datasets by capturing dimensions of human experience that cannot be adequately reflected in quantitative environmental indicators. All datasets were standardized after collection to ensure the scientific rigor and comparability of the quantitative analyses.

The questionnaire survey involved residents’ subjective perception data, participation was entirely voluntary and anonymous. Informed consent was obtained from all participants through a statement on the first page of the questionnaire, and additional oral explanations were provided during on-site distribution to ensure participants’ full understanding and agreement.

### 2.3 Methods

This study establishes a comprehensive evaluation system for the health restorative potential of waterfront parks by integrating spatial analysis, statistical analysis, and questionnaire surveys. The methodological framework encompasses five stages: extraction of restorative environmental factors, subjective evaluation of indicators, objective analysis of indicators, determination of weights, and comprehensive evaluation with result interpretation. Specifically, the study employs kernel density analysis, questionnaire design, PRS scale assessment, principal component analysis, correlation analysis, mean value analysis, regression analysis, and a comprehensive weighting approach to construct a multidimensional, multi-source evaluation model.

#### 2.3.1 Kernel density estimation (KDE)

Kernel density estimation is a spatial statistical method used to estimate the distribution characteristics of point data by calculating the density of points around each location, thereby generating a continuous density surface to reveal spatial hotspots and distribution patterns. In this study, KDE was applied to assess the spatial density distribution of restorative environmental factors within and around the parks, thereby identifying core clustering zones of environmental attributes and providing spatial evidence for the subsequent selection of evaluation indicators. The general formula of KDE is:


$$f(x)=\frac{1}{\mathrm{nh}}\sum_{i=1}^{n}K(\frac{x-x_{i}}{h})$$

Where: f(x) is the estimated density at location x; n is the number of sample points; h is the bandwidth (smoothing parameter); K is the kernel function; $x_{i}$ is the observed data points.

#### 2.3.2 Questionnaire design

The questionnaire survey served as the primary tool for collecting first-hand data on participants’ subjective perceptions and evaluations. It consisted of three parts: (1) Demographic information: including age, education, and occupation, used to distinguish group preferences and evaluation differences. (2) Environmental factor satisfaction: direct assessment of 28 environmental factors across the seven waterfront parks, measured using a five-point Likert scale (1 = very dissatisfied, 5 = very satisfied). (3) Perceived Restorativeness Scale (PRS): adapted from Hartig’s PRS, employing a Likert scale to evaluate the restorative features of parks from four dimensions-Being Away, Fascination, Compatibility, and Extent. The PRS in this study consisted of 11 items, such as “This place is fascinating,” “Being here allows me to get away from daily hassles,” and “The setting is well organized,” which collectively measure the perceived restorativeness of the park environment across the four dimensions. The comprehensive PRS score was calculated as:


$$\mathrm{PRS}=\frac{\sum_{i=1}^{m}S_{i}}{m}$$

Where: PRS is the overall perceived restorativeness score; $S_{i}$ is the score of indicator i; m is the total number of PRS items. These data provided subjective insights into the psychological dimension of restorative experiences in the selected waterfront parks.

#### 2.3.3 Principal component analysis (PCA)

Principal component analysis is a multivariate statistical technique for dimensionality reduction. It transforms correlated variables into a smaller set of uncorrelated composite variables (principal components) that capture the majority of the variance in the data, thus minimizing redundancy and emphasizing key information. In this study, PCA was applied to multiple objective indicators under three health dimensions-psychological, physiological, and social interaction-to extract core composite factors reflecting the restorative characteristics of waterfront parks and to mitigate multicollinearity in subsequent evaluations. The general PCA formulation is:


$$Z_{j}=a_{j1}X_{1}+a_{j2}X_{2}+\ldots +a_{\mathrm{jp}}X_{p}$$

Where: $Z_{j}$ is the j-th principal component; $X_{p}$ is the original variables; $a_{\mathrm{jp}}$ is the loading coefficient of variable $X_{p}$ on component $Z_{j}$.

#### 2.3.4 Correlation analysis

Correlation analysis measures the degree of linear association between two variables, typically using Pearson’s correlation coefficient. In this study, correlation analysis was conducted to detect redundancy among health-related indicators, ensuring that highly correlated variables were not repeatedly weighted, thereby improving the parsimony and robustness of the evaluation framework.


$$r=\frac{\sum (X_{i}-\bar{X})(Y_{i}-\bar{Y})}{\sqrt{\sum {\left(X_{i}-\bar{X}\right)}^{2}\sum {\left(Y_{i}-\bar{Y}\right)}^{2}}}$$

Where: r is the Pearson correlation coefficient; $X_{i},Y_{i}$ is the paired observations of variables X and Y; $\bar{X},\bar{Y}$ is the means of X and Y.

#### 2.3.5 Mean value analysis

Mean value analysis compares the average levels of indicators across different study objects, enabling horizontal comparisons of performance differences. In this study, mean value analysis was employed to evaluate the differences in psychological, physiological, and social interaction health factors across the seven waterfront parks, highlighting their relative strengths and weaknesses and providing empirical evidence for classification and optimization strategies. The general formula is:


$$\bar{X}=\frac{1}{n}\sum_{i=1}^{n}X_{i}$$

Where: $\bar{X}$ is the mean value of indicator X; n is the number of observations; $X_{i}$ is value of indicator X in observation i.

#### 2.3.6 Regression analysis

Regression analysis investigates the relationship between a dependent variable and one or more independent variables, establishing a predictive or explanatory model. In this study, multiple regression was applied to examine the contribution of health-related indicators to the restorative index, thereby identifying key variables that significantly affect the health-restorative function of waterfront parks. The multiple linear regression model is expressed as:


$$Y=\beta_{0}+\beta_{1}X_{1}+\beta_{2}X_{2}+\ldots +\beta_{p}X_{p}+\varepsilon$$

Where: Y is the dependent variable (restorative index); $X_{p}$ is the independent variables (health-related indicators); $\beta_{0}$ is the constant term; $\beta_{p}$ is the regression coefficients; ε is the error term.

#### 2.3.7 Integrated weighting method

The integrated weighting method combines subjective and objective weighting approaches to reduce potential bias from a single method. In this study, the analytic hierarchy process (AHP) and the entropy weight method were applied. First, expert judgment was used to construct pairwise comparison matrices and derive subjective weights via AHP. Then, entropy values of each indicator were calculated to obtain objective weights. Finally, the two were integrated proportionally to determine comprehensive weights that reflect both data characteristics and expert knowledge. The comprehensive weight formula is:


$$W_{j}=\alpha W_{j}^{\mathrm{AHP}}+(1-\alpha )W_{j}^{\text{Entropy}}$$

Where: $W_{j}$ is the final comprehensive weight of indicator j; $W_{j}^{\mathrm{AHP}}$is the weight of indicator j from AHP; $W_{j}^{\text{Entropy}}$ is the weight of indicator j from entropy method; α is the adjustment coefficient balancing subjective and objective weights. The value of α was set to 0.5, based on expert consultation and argumentation with professors and associate professors in the field of urban planning (49, 50).

## 3 Results

### 3.1 Restorative environmental factors in waterfront parks

#### 3.1.1 Survey on restorative behaviors

**Figure 3:**
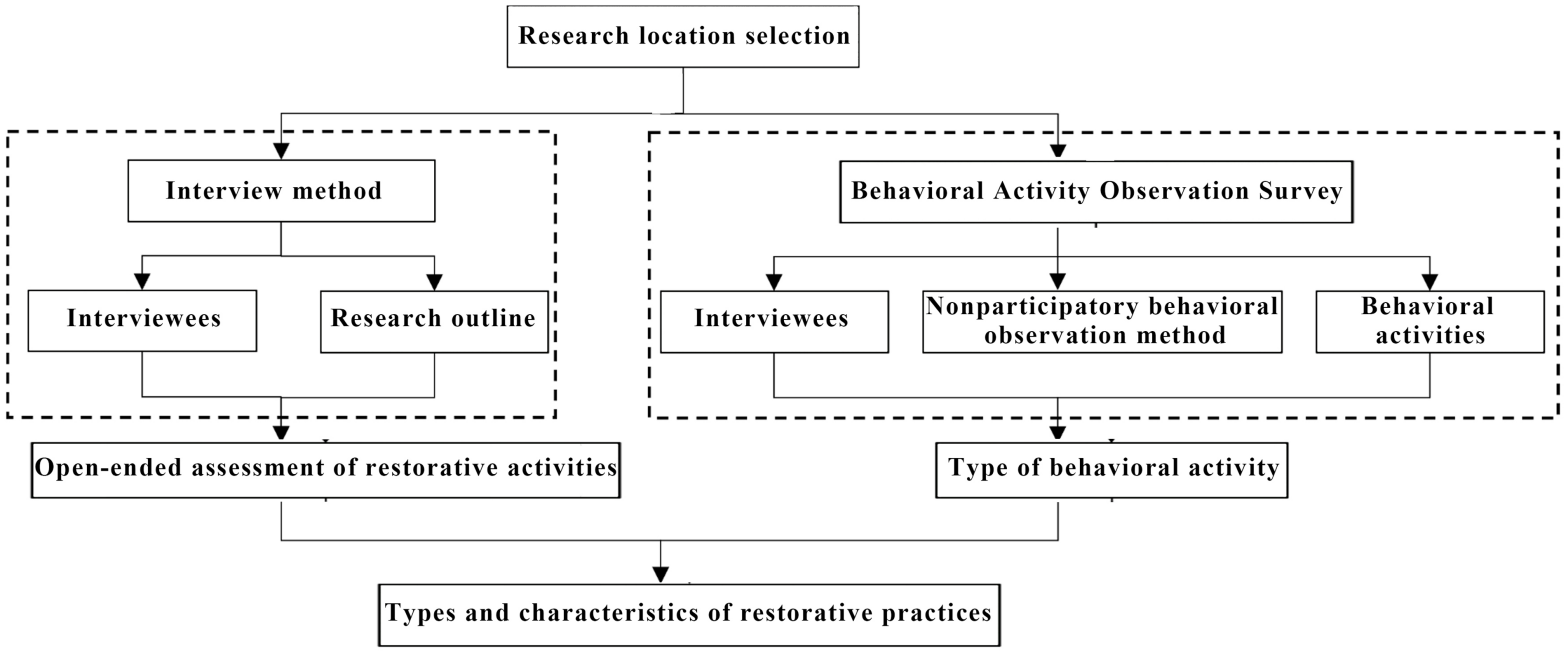
Research process flowchart.

To systematically identify the types and characteristics of residents’ restorative activities in waterfront parks, this study conducted field investigations in two phases across seven representative waterfront parks along the Hun River (Figure 3). Phase I involved an open-ended questionnaire survey, designed to collect residents’ self-reported preferences for health-oriented restorative activities. Phase II consisted of behavioral observation surveys, aimed at recording and quantifying the actual types of activities and their spatial distribution within the parks.

##### 3.1.1.1 Open-ended questionnaire survey

Before the formal distribution of questionnaires, a pilot survey involving 30 randomly selected participants was conducted to verify the clarity and reliability of the items. The pilot responses achieved a 100% recovery rate, and the Cronbach’s Alpha coefficient was 0.919, indicating high internal consistency. Based on the feedback from this pre-test, the questionnaire was refined and finalized.

From September 10 to 11, 2022, the research team conducted on-site open-ended interviews in seven selected waterfront parks: Luoshichuan Ecological Park, Shenshuiwan Park, Wulihe Park, Changbai Island Forest Park, Heping Sports Park, Hunnan Citizen Park, and Olympic Park. Subsequently, 70 questionnaires were formally distributed (10 in each park) to frequent park users, resulting in 64 valid responses and a response rate of 91.4%. The reliability tests of the final questionnaire and the restorative scale yielded Cronbach’s Alpha coefficients of 0.922 and 0.958, respectively, both exceeding 0.9, which demonstrates excellent reliability. Although the total sample size is relatively small, the targeted sampling approach enables precise assessment of typical user experiences within each park, aligning with the principle of small-sample, context-specific research.

The central question was: *“What activities would you prefer to engage in within the park to restore your physical and mental health?.”* Results (Table 1) showed that “strolling in the park” was the most frequently mentioned activity (25%). However, this category encompassed multiple sub-activities, including resting, walking, cycling, fishing, playing, dog-walking, and exercising. Based on spatial location, implementation mode, and behavioral characteristics, combined with high-frequency word analysis, the self-reported restorative activities were classified into five categories: fitness, recreational, specialized, social, and quiet activities (Table 1). This classification provides empirical evidence for identifying restorative behavioral components in waterfront parks.

**Table 1:** Summary of types of restorative activities.

Restore behavioral activity categories	Types of restorative activities
Fitness	Ball games, square dancing, fitness equipment, running, cycling, etc.
Entertainment	Playing cards, chess, etc.
Specialized	Kite flying, fishing, dog walking, etc.
Social	Parties, picnics, camping, etc.
Quiet	Reading, sunbathing, meditation, resting, etc.

##### 3.1.1.2 Behavioral observation

To validate the consistency between self-reported and actual behaviors, and to obtain objective data on activity distribution, non-participatory field observations were carried out across the seven parks. Residents’ specific activities were recorded across different time periods. The results indicated that walking was the most prevalent activity, reaching 36% in Heping Sports Park and 25% in Luoshichuan Ecological Park. Running was particularly prominent in Changbai Island Forest Park (8%) and Hunnan Citizen Park (7.2%). Facility-related activities (e.g., outdoor gym equipment, ball games, and recreational facilities) were more frequent in Shenshuiwan Park (fitness 12%, ball games 9%) and Wulihe Park (fitness 8.9%, recreation 8.1%, and ball games 6.1%). Distinctive activities were also observed: skateboarding and roller-skating in Wulihe Park, grass-sitting in Heping Sports Park (10%, the highest among all parks), and free activities in Olympic Park (16.6%).

**Figure 4:**
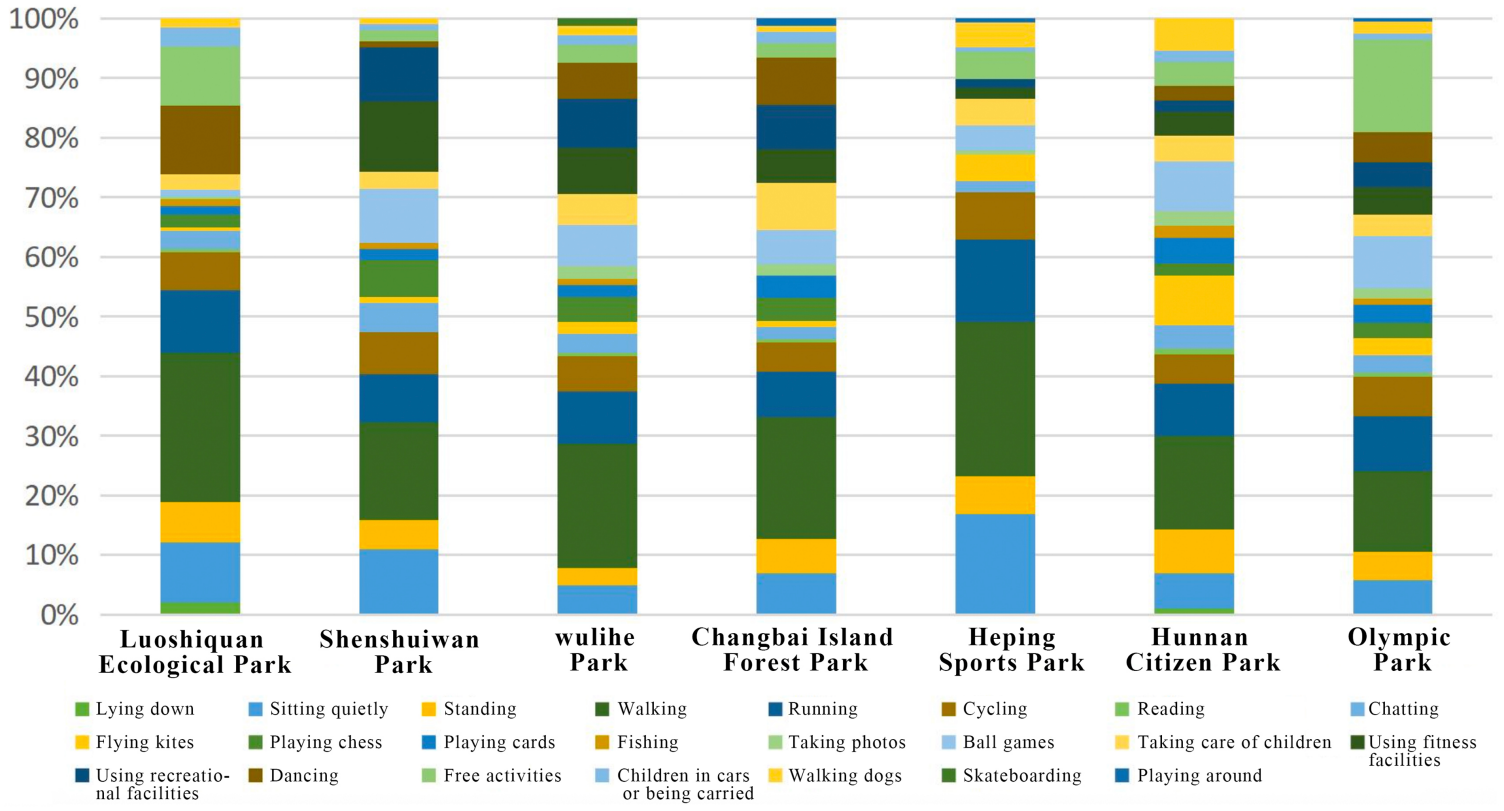
Activity statistics for each park’s recovery.

Overall, 23 types of activities were recorded (Figure 4). Among them, walking (20.19%), running (17.16%), and facility-related activities (14.48%) ranked as the top three. Dynamic activities such as walking, running, and cycling together accounted for 43.26%. Notably, these activities were primarily concentrated along riverfront trails, highlighting the central role of linear waterfront spaces in facilitating restorative behaviors.

##### 3.1.1.3 Integrated analysis of survey results

A comparative analysis of the open-ended questionnaires and field observations demonstrated that residents’ self-reported activities largely aligned with actual observed behaviors, with no evidence of destructive or antisocial activities. Consequently, these activities were collectively defined as *restorative behaviors*. Integrating the five categories derived from the open-ended survey with the 23 observed activity types, and considering the restorative needs of different user groups, the restorative activities in waterfront parks were ultimately classified into three overarching types: psychological restoration, physiological restoration, and social interaction (Table 2). This behavioral foundation informs the subsequent construction and evaluation of restorative environmental factors.

**Table 2:** Summary table of restorative behavior activity categories.

Restoring demand levels	Restoring demand characteristics	Restoring modes of action	Restoring behavioral content	Restoring behavioral characteristics
Mental health recovery	Depression, restlessness, tension, anxiety, etc.	Improve mental health	Enjoying natural scenery, watching water features, sitting quietly, thinking, reading, etc.	Get in touch with nature, relax your body and mind
Physical health recovery	Physical weakness, poor endurance, cardiovascular disease, obesity, etc.	Promote physical activity and ecological health benefits	Aerobic exercises such as running and cycling; ball games, weight training, etc.	Focus on activities such as facility sports and field sports
Social health recovery	Social isolation, poor communication skills with others	Increase communication and interaction among urban residents	Parties, picnics, camping, card games, square dancing, socializing with other mothers, etc.	Focus on group activities

#### 3.1.2 Extraction of restorative environmental factors

Building upon the categorization of restorative behaviors in waterfront parks presented in the previous section, this part further analyzes the spatial distribution patterns of different restorative behaviors to extract the environmental attributes of their occurrence sites. These attributes serve as empirical evidence for the subsequent construction of the evaluation indicator system.

First, based on on-site survey data of residents’ restorative behavior points, the observed activity types and locations were imported into the ArcGIS platform. Kernel Density Estimation (KDE) was applied to weekday and weekend datasets separately to reveal spatial clustering patterns of restorative behaviors. The degree of clustering reflects the attractiveness and preference of spatial use: the higher the clustering intensity, the greater the potential of the space for restorative use.

To ensure data accuracy and traceability, a “cat-eye quadrant” recording tool was used during field surveys to capture and geolocate residents’ restorative behaviors, with photographic evidence preserved for supplementary verification. Secondary field inspections were conducted at high-density clusters identified by KDE, where environmental attributes such as spatial morphology, landscape features, facility configuration, and surrounding conditions were systematically documented.

The main spatial characteristics of high-density clusters in each park are summarized as follows (Table 3):

(1) Luoshichuan Ecological Park: Two clusters were identified, both in open plaza spaces. The core plaza covers ~0.8 ha, with a clear north–south visual corridor and functions as both a landmark landscape and activity venue. The other cluster is a small plaza with a pavilion, primarily supporting rest and social interactions.
(2) Shenshuiwan Park: Four clusters were observed, including a riverside trail with a cultural node (Shanmen Temple), multifunctional sports grounds, a centralized fitness zone, and an under-bridge free activity space. Together, these areas support psychological, physiological, and social restorative needs.
(3) Wulihe Park: Two clusters were located in plaza-type spaces (Qinkai Plaza and another multifunctional square), characterized by open views and high functional diversity.
(4) Changbai Island Forest Park: Two clusters were observed, located in an under-bridge sports ground and an open space within the park. The former benefits from climatic shelter and multiple facilities, while the latter supports static rest and board-game activities.
(5) Heping Sports Park: Two clusters with similar attributes were identified (Lang Lang Piano Plaza and its adjacent area), enclosed by trees, with undulating topography and ample sunlight, offering combined psychological, physiological, and social restorative potential.
(6) Hunnan Citizen Park: A main cluster was located near a badminton court and open waterfront area, where camping and youth social gatherings are concentrated.
(7) Olympic Park: Two clusters were identified: a riverside plaza adjacent to urban roads, well-equipped with rest facilities, and a highly connected landscape trail, characterized by diverse vegetation and suitable for cycling.

**Table 3:** The main spatial characteristics of restorative activities in various parks.

Park	Working day	Rest day	Gathering point	Feature summary	Activity type
Luoshichuan Ecological Park	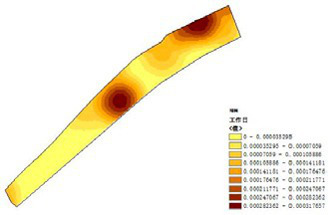	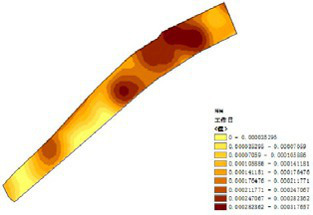	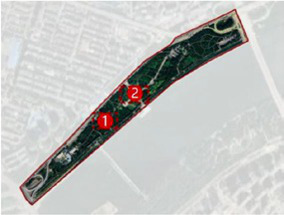	High openness, rest areas, activity areas, landmark structures	Meditation, chatting, flying kites, tai chi
Shenshuiwan Park	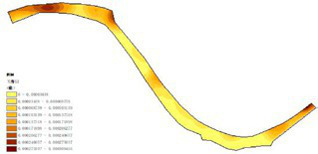	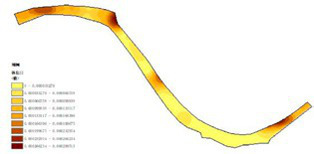	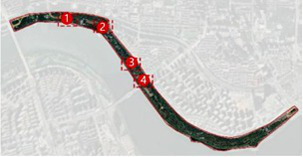	Many types of plants, Open space, Wide variety of activity facilities, Unobstructed view	Walking, running, cycling, playing table tennis, playing chess, using fitness equipment, etc.
Wulihe Park	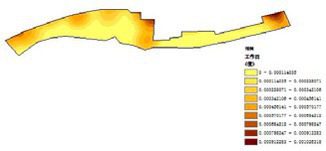	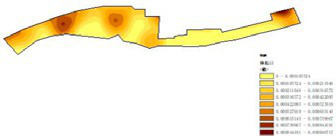	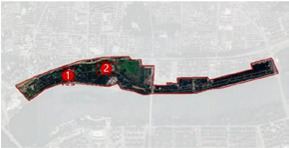	High openness, Open space, Structures, Rich colors	Flying kites, skateboarding, rollerblading, etc.
Changbai Island Forest Park	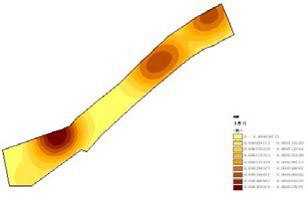	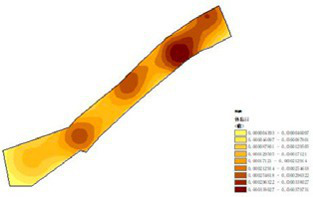	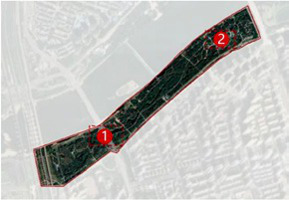	Highly functional, spacious grounds, diverse plant species, rest areas	Basketball, Soccer, Table tennis, Square dancing, chess Meditation, etc.
Heping Sports Park	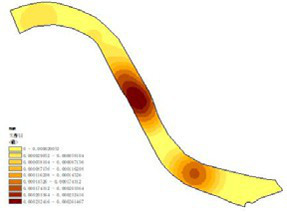	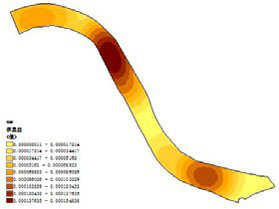	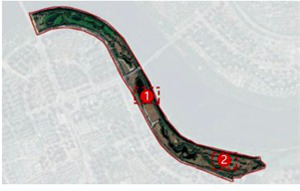	Large venue area Unobstructed view	Meditation, kite flying, picnics, camping, tai chi, and play.
Hunnan Citizen Park	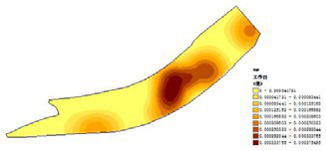	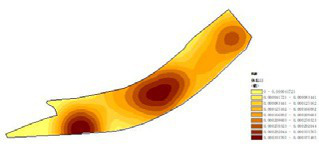	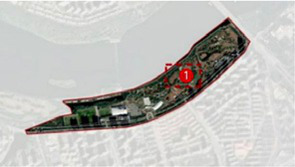	Open view, Close to waterways	Camping, Flying kites, Walking dogs
Olympic Park	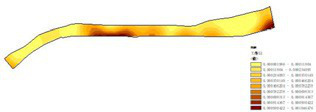	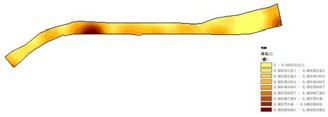	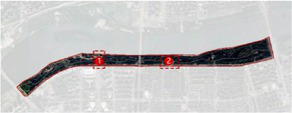	Wide view, waterfront platform, open space, numerous rest facilities, wide variety of activity facilities	Meditation, Playing in the water, Using fitness equipment, Walking, Running, Cycling

After systematically documenting the environmental characteristics of existing restorative behavior spaces, and integrating insights from the restorative environment theoretical framework and related studies, a total of 21 evaluation factors influencing the health-restorative potential of waterfront parks were extracted (Table 4). These include:

Visual and landscape attributes: visual colorfulness, revetment type, water landscape attractiveness, vegetation type, green view index, vegetation coverage, topography, waterfront accessibility, and visual openness.

Perceptual and environmental attributes: perceived spatial safety, spatial privacy, environmental quietness, environmental sanitation, and external traffic disturbance.

Facility and spatial attributes: diversity and quantity of recreational/fitness facilities, quantity and quality of ornamental facilities, activity space area, slow-mobility network density, internal path connectivity, accessibility facilities completeness, number and comfort of resting facilities, road intersection density, road density, public transport facility density, and number of park entrances.

**Table 4:** Restorative impact factors of waterfront parks and their sources.

Indicator name	Indicator source	Restorative environmental characteristics
Visual colorfulness	References	Being away, fascination, extent, compatibility
Vegetation type	Theoretical research	Fascination, compatibility
Vegetation coverage	References	Being away, fascination, extent
Environmental quietness	Theoretical research	Being away, fascination, extent
Environmental hygiene	Theoretical research	Fascination, compatibility
Reinforced bank type	Theoretical research	Fascination, compatibility
Water scenery	Theoretical research	Fascination
Topography	Theoretical research	Fascination, extent
Spatial sense of security	Theoretical research	Being away
Spatial privacy	Theoretical research	Being away, extent
Open view	Current status survey	Being away, fascination, extent
Green view ratio	References	Being away, fascination, extent
Road connectivity	Theoretical research	Fascination, extent
Slow traffic network density	Theoretical research	Extent
Surrounding traffic interference	References	Being away, extent
Types of entertainment and fitness facilities	Theoretical research	Being away, extent
Number of entertainment and fitness facilities	Theoretical research	Being away, extent
Number of viewing facilities	References	Being away, fascination, extent
Quality of viewing facilities	References	Being away, fascination, extent
Activity area	Theoretical research	Being away, extent
Number of rest facilities	Theoretical research	Being away, extent

#### 3.1.3 Correlation analysis between restorative effects and environmental factors

**Figure 5:**
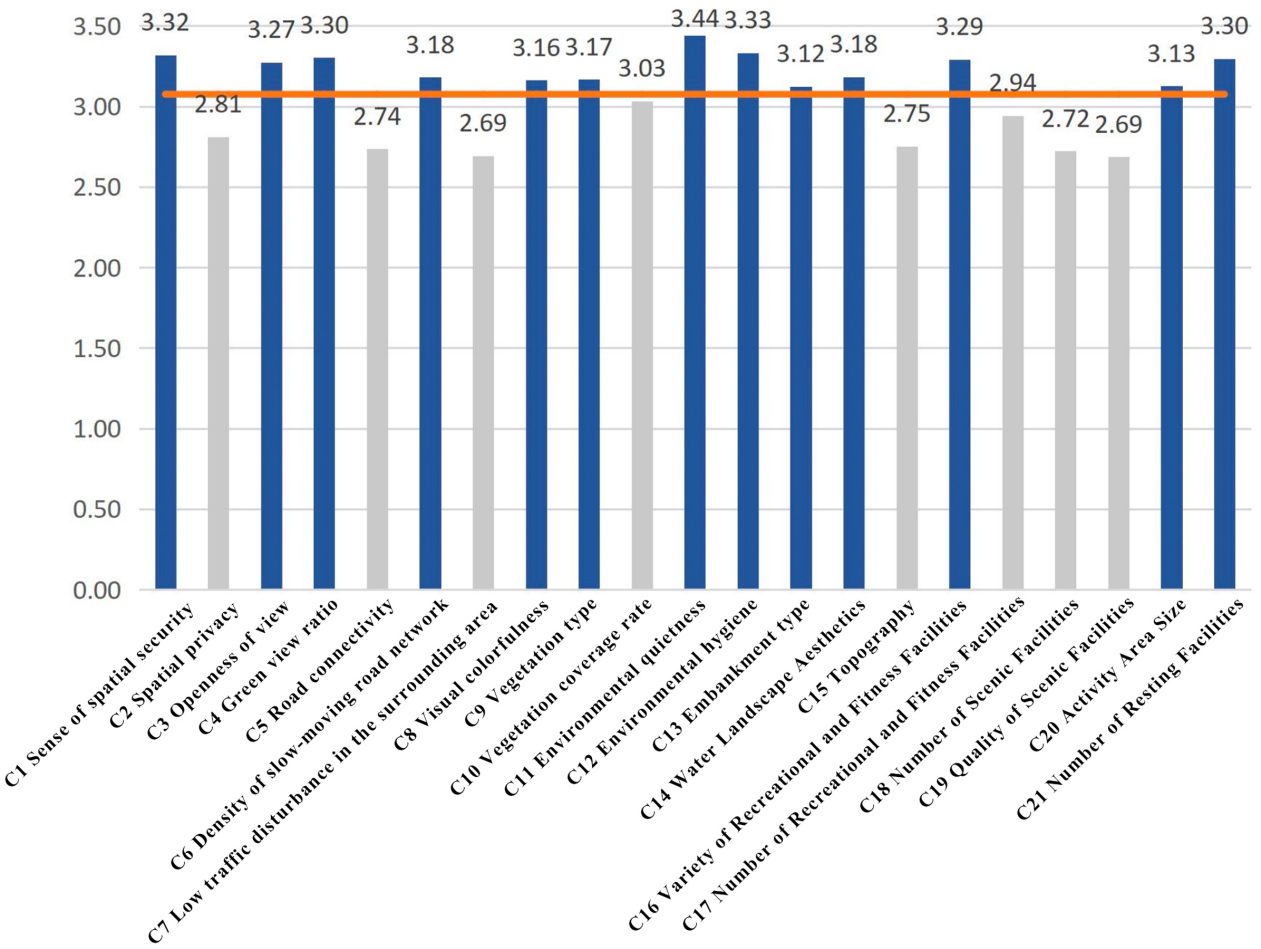
Analysis of mean values for each factor.

(1) Principal component analysis (PCA): To identify the underlying structure among environmental factors and to achieve dimensionality reduction, PCA with varimax rotation was applied to the pre-selected 21 environmental variables. The results (Table 5) revealed three principal components with eigenvalues of 12.450, 3.223, and 2.170, accounting for a cumulative variance of 80.95%, which meets the requirements of both dimensionality reduction and explanatory power.
(2) Based on factor loadings (Table 6), the original variables were classified into three groups: (i) Physiological health-related factors, reflecting natural elements such as vegetation, water, and topography (including visual colorfulness, vegetation type and coverage, environmental quietness, sanitation, revetment type, water landscape attractiveness, and); (ii) Psychological health-related factors, emphasizing subjective perception and spatial experience (including perceived spatial safety, privacy, visual openness, green view index, road connectivity, slow-mobility network density, and traffic disturbance); and (iii) Social interaction-related factors, focusing on activity and facility provision (including diversity and quantity of recreational/fitness facilities, quantity and quality of ornamental facilities, activity space area, and resting facilities). This three-factor structure aligns with the “nature–perception–behavior” framework of Attention Restoration Theory, providing both theoretical and empirical support for subsequent analyses.
(3) Mean value analysis: A descriptive mean analysis of the 21 factors was conducted to capture their overall performance trends (Figure 5). The average score across all factors was approximately 3.07. Several variables-such as spatial safety, visual openness, green view index, slow-mobility density, visual colorfulness, vegetation type, environmental quietness, sanitation, revetment type, water landscape attractiveness, diversity of recreational facilities, activity space area, and resting facility quantity-scored above the mean, indicating generally favorable conditions or strong public perceptions in the study area. In contrast, variables such as spatial privacy, road connectivity, vegetation coverage, quantity of recreational/fitness facilities, quantity and quality of ornamental facilities, and topography scored below the mean. It should be emphasized that mean values only reflect general trends and cannot determine causality or significance with restorative outcomes. Hence, mean analysis was employed as a preliminary screening tool, followed by correlation and regression analysis for empirical validation.
(4) Correlation analysis (Table 7): Pearson correlation coefficients were used to evaluate the strength of association between environmental factors and residents’ self-reported restorative outcomes. According to conventional thresholds, |*r*| < 0.2 indicates negligible correlation, 0.2 ≤ |*r*| < 0.4 weak correlation, 0.4 ≤ |*r*| < 0.6 moderate correlation, and |*r*| ≥ 0.6 strong correlation. Results showed that most natural–spatial–facility indicators-including spatial safety, privacy, visual openness, green view index, road connectivity, slow-mobility density, vegetation type and coverage, environmental quietness and sanitation, revetment type, water landscape attractiveness, diversity and quantity of recreational/fitness facilities, quantity and quality of ornamental facilities, activity space area, and resting facilities-were positively correlated with restorative outcomes. A few indicators (e.g., visual colorfulness) exhibited weak negative or statistically non-significant correlations, while traffic disturbance and topography showed non-significant correlations. Overall, the correlation analysis confirmed widespread positive associations between candidate factors and restorative effects, providing a basis for regression modeling and variable selection.
(5) Regression analysis (Table 8): Following Pearson correlation analysis, variables with coefficients below 0.2 (e.g., traffic disturbance and topography) were excluded, and multiple linear regression was performed on the remaining significant variables to identify dominant influencing factors. The regression model yielded an adjusted *R*^2^ of 0.631, indicating that the selected environmental variables explain approximately 63.1% of the variance in restorative outcomes. Variance Inflation Factors (VIFs) were all below 5, suggesting no serious multicollinearity. Standardized regression coefficients indicated that spatial privacy (*β* ≈ 0.55), quantity of recreational/fitness facilities (*β* ≈ 0.46), revetment type (*β* ≈ 0.44), water landscape attractiveness (*β* ≈ 0.44), visual openness (*β* ≈ 0.41), and spatial safety (*β* ≈ 0.41) were the strongest predictors of restorative outcomes. Secondary factors included environmental sanitation, diversity of recreational facilities, quantity of resting facilities, green view index, and quality of ornamental facilities (*β* between 0.2 and 0.4). Other variables had weaker effects (*β* < 0.2). These findings suggest that both objective natural features (shoreline and water landscape) and subjective spatial perceptions (privacy, openness, and safety) jointly determine the restorative efficacy of waterfront parks.
(6) Weight determination (Table 9): Based on the PCA and regression results, standardized regression coefficients were used as the basis for determining the relative influence of individual indicators, which were then aggregated into weights for the three major factor categories. The results showed that psychological health factors (dominated by spatial privacy, openness, and safety) carried the highest weight, followed by physiological health factors (dominated by water landscape attractiveness, revetment type, and vegetation), and finally social interaction health factors (dominated by recreational facilities and activity spaces). This weighting scheme not only reflects the statistical explanatory power but also has clear planning implications: to enhance the restorative potential of waterfront parks, priority should be given to improving spatial perception quality (privacy, safety, and openness), while simultaneously strengthening natural elements (waterfront and vegetation) and supporting them with adequate activity facilities to meet social interaction needs.

**Table 5:** Total variance explanation.

Component	Initial eigenvalue			Extracting the sum of squared loads			The sum of rotating load		
	Total	Percentage variance	Accumulation	Total	Percentage variance	Accumulation	Total	Percentage variance	Accumulation
1	12.45	47.89	47.89	12.45	47.89	47.89	5.12	19.71	19.71
2	3.22	12.40	60.28	3.22	12.40	60.28	4.10	25.77	45.48
3	1.97	7.58	67.86	2.17	7.58	67.86	4.02	35.47	80.95
4	1.70	6.54	74.40						
5	1.40	5.40	79.80						
6	0.55	2.13	81.93						
7	0.50	1.90	83.83						
8	0.47	1.80	85.63						
9	0.39	1.49	87.12						
10	0.37	1.42	88.54						
11	0.34	1.29	89.83						
12	0.32	1.24	91.06						
13	0.28	1.09	92.16						
14	0.28	1.07	93.22						
15	0.25	0.95	94.17						
16	0.24	0.91	95.08						
17	0.20	0.78	95.85						
18	0.18	0.70	96.55						
19	0.17	0.67	97.22						
20	0.16	0.61	97.83						
21	0.15	0.57	98.40						

**Table 6:** Rotating component load matrix.

Indicator number	Name of indicator	Component		
		1	2	3
C1	Sense of spatial security	0.151	0.246	0.709
C2	Spatial privacy	0.146	0.141	0.744
C3	Openness of view	0.162	0.182	0.751
C4	Green view ratio	0.173	0.341	0.673
C5	Road connectivity	0.126	0.322	0.712
C6	Density of slow-moving road network	0.144	0.122	0.769
C7	Low traffic disturbance in the surrounding area	0.162	0.122	0.681
C8	Visual colorfulness	0.635	0.193	0.325
C9	Vegetation type	0.932	0.187	0.196
C10	Vegetation coverage rate	0.833	0.165	0.265
C11	Environmental quietness	0.811	0.168	0.250
C12	Environmental hygiene	0.852	0.144	0.273
C13	Embankment type	0.895	0.128	0.216
C14	Water landscape aesthetics	0.881	0.322	0.335
C15	Topography	0.615	0.122	0.094
C16	Variety of recreational and fitness facilities	0.173	0.823	0.151
C17	Number of recreational and fitness facilities	0.163	0.784	0.175
C18	Number of scenic facilities	0.271	0.767	0.143
C19	Quality of scenic facilities	0.117	0.772	0.129
C20	Activity area size	0.142	0.799	0.151
C21	Number of resting facilities	0.212	0.806	0.156

*The asterisk (*) following the number indicates the degree of significance in factor correlations. * denotes significant, ** denotes moderately significant, and *** denotes highly significant.

**Table 7:** Correlation analysis.

Factor class		Name of indicator	Correlation coefficient	*p* value
Mental health impact factors	C1	Sense of spatial security	0.203*	0.029
	C2	Spatial privacy	0.706***	0.265
	C3	Openness of view	0.739***	0
	C4	Green view ratio	0.420**	0.002
	C5	Road connectivity	0.321*	0.019
	C6	Density of slow-moving road network	0.581**	0.004
	C7	Low traffic disturbance in the surrounding area	0.096	0.543
	C8	Visual colorfulness	−0.279*	0.042
Physiological health Impact factors	C9	Vegetation type	0.519**	0.008
	C10	Vegetation coverage rate	0.368*	0.007
	C11	Environmental quietness	0.383**	0
	C12	Environmental hygiene	0.402**	0.002
	C13	Embankment type	0.695***	0.031
	C14	Water landscape aesthetics	0.657***	0.006
	C15	Topography	0.168	0.429
Social interaction Impact factors	C16	Variety of recreational and fitness facilities	0.528***	0
	C17	Number of recreational and fitness facilities	0.669***	0
	C18	Number of scenic facilities	0.375*	0.021
	C19	Quality of scenic facilities	0.267*	0.039
	C20	Activity area size	0.670***	0
	C21	Number of resting facilities	0.417**	0.001

* denotes significant, ** denotes moderately significant, and *** denotes highly significant.

**Table 8:** Regression analysis of environmental factors of waterfront park.

Indicator number	Name of indicator	Non-standardized coefficient		Standardization coefficient	VIF value
C1	Sense of spatial security	0.036	0.131	0.409	2.274
C2	Spatial privacy	−0.232	0.142	0.548	1.349
C3	Openness of view	0.481	0.124	0.413	3.991
C4	Green view ratio	0.528	0.122	−0.229	2.123
C5	Road connectivity	−0.054	0.139	−0.054	3.902
C6	Density of slow-moving road network	0.557	0.121	0.202	2.513
C7	Visual colorfulness	0.092	0.129	0.094	1.821
C8	Vegetation type	−0.020	0.156	−0.026	4.675
C9	Vegetation coverage rate	−0.042	0.164	−0.042	3.087
C10	Environmental quietness	−0.104	0.146	−0.097	3.517
C11	Environmental hygiene	0.329	0.268	0.340	1.618
C12	Embankment type	0.328	0.110	0.439	2.414
C13	Water landscape aesthetics	0.037	0.131	0.437	3.655
C14	Variety of recreational and fitness facilities	0.267	0.091	0.278	2.561
C15	Number of recreational and fitness facilities	−0.420	0.120	−0.457	3.741
C16	Number of scenic facilities	0.141	0.141	0.152	1.663
C17	Quality of scenic facilities	0.205	0.143	0.215	4.307
C18	Activity area size	0.081	0.139	0.091	2.281
C19	Number of resting facilities	0.260	0.135	0.264	1.468

**Table 9:** Weight of each indicator.

Target layer		Weight	Criterion layer		Weight	Indicator number	Index layer	Weight
A1	Waterfront Park health recovery	1	B1	Mental health impact factors	0.419	C1	Sense of spatial security	0.045
						C2	Spatial privacy	0.107
						C3	Openness of view	0.062
						C4	Green view ratio	0.057
						C5	Road connectivity	0.035
						C6	Density of slow-moving road network	0.041
						C7	Visual colorfulness	0.072
			B2	Physiological health impact factors	0.265	C8	Vegetation type	0.035
						C9	Vegetation coverage rate	0.029
						C10	Environmental quietness	0.027
						C11	Environmental hygiene	0.016
						C12	Embankment type	0.067
						C13	Water landscape aesthetics	0.091
			B3	Social interaction impact factors	0.316	C14	Variety of recreational and fitness facilities	0.091
						C15	Number of recreational and fitness facilities	0.082
						C16	Number of scenic facilities	0.023
						C17	Quality of scenic facilities	0.037
						C18	Activity area size	0.081
						C19	Number of resting facilities	0.041

### 3.2 Evaluation of health restorativeness in Shenyang’s Hunhe riverside parks

In the previous section, data analysis clarified the evaluation indicator system for health restorativeness in riverside parks and determined the weight of each indicator, thereby establishing a comprehensive evaluation framework. This chapter conducts a specific health restorativeness evaluation for the parks within the study area. By quantitatively analyzing the indicators of each park, it comprehensively examines their performance in terms of psychological health, physiological health, and social interaction, and further assesses their restorative effects. Ultimately, based on the commonalities and differences among the parks, it identifies the key optimization directions for different park types, thus providing scientific evidence for enhancing the health restorativeness of riverside parks.

#### 3.2.1 Indicator quantification and evaluation model construction

The indicator quantification methods in this chapter are divided into subjective and objective categories. Objective indicators are mainly obtained through web data crawling, field surveys, and spatial analysis to ensure objectivity and accuracy. Subjective indicators are derived from questionnaire survey data to supplement aspects of public perception and experience not captured by objective data.

Through the quantitative analysis of objective indicators in psychological, physiological, and social interaction health factors, this chapter provides a quantitative evaluation of the restorative effect of each park. The results are then visualized to present indicator performance, laying the foundation for subsequent comprehensive evaluation and optimization recommendations.

##### 3.2.1.1 Mental health influence factors

**Figure 6:**
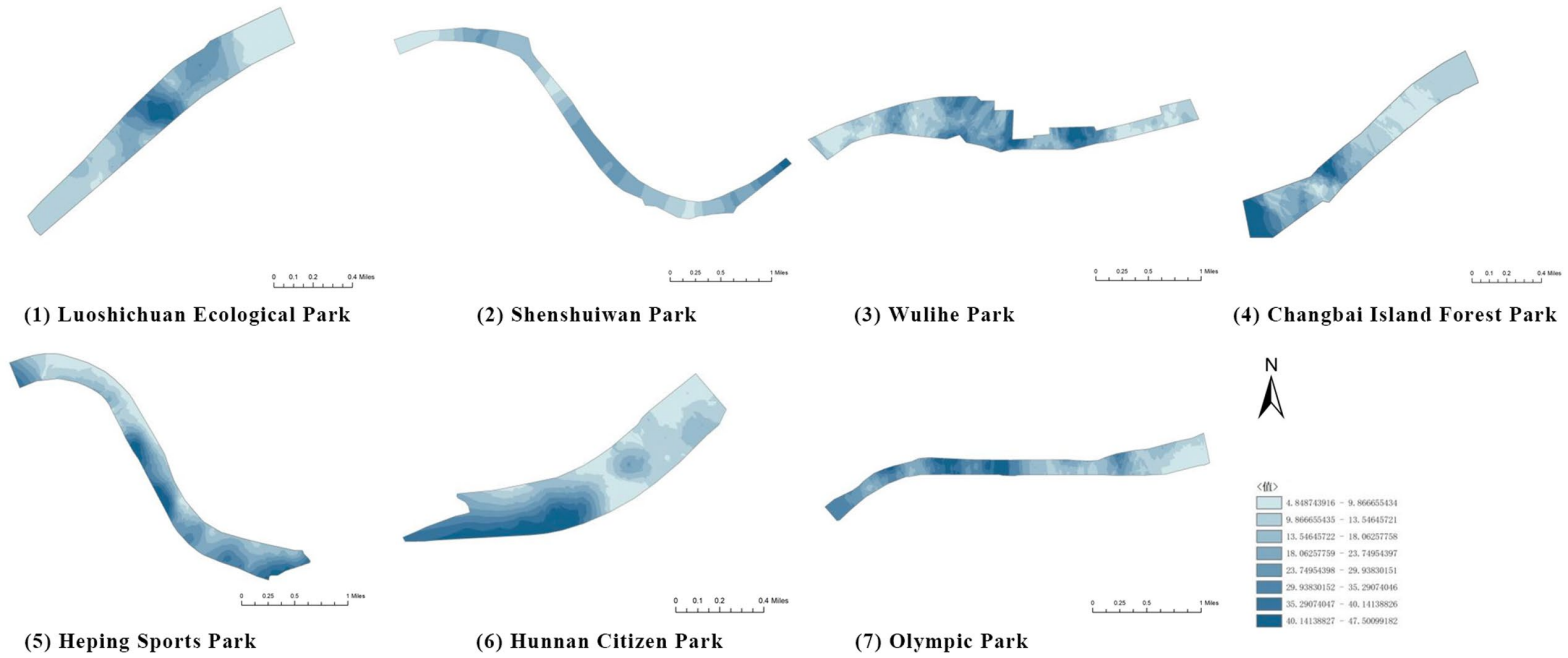
Spatial openness of each park.

**Figure 7:**
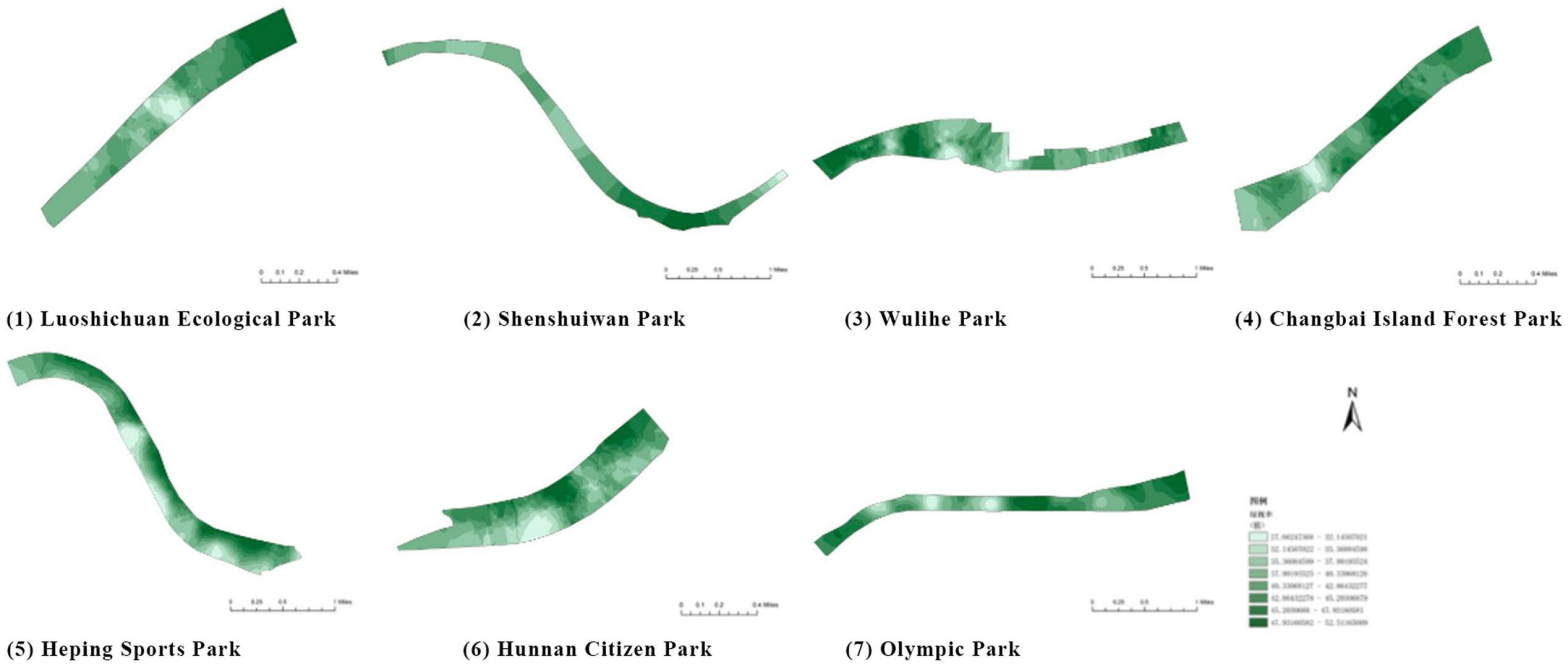
Green view index of each park.

**Figure 8:**
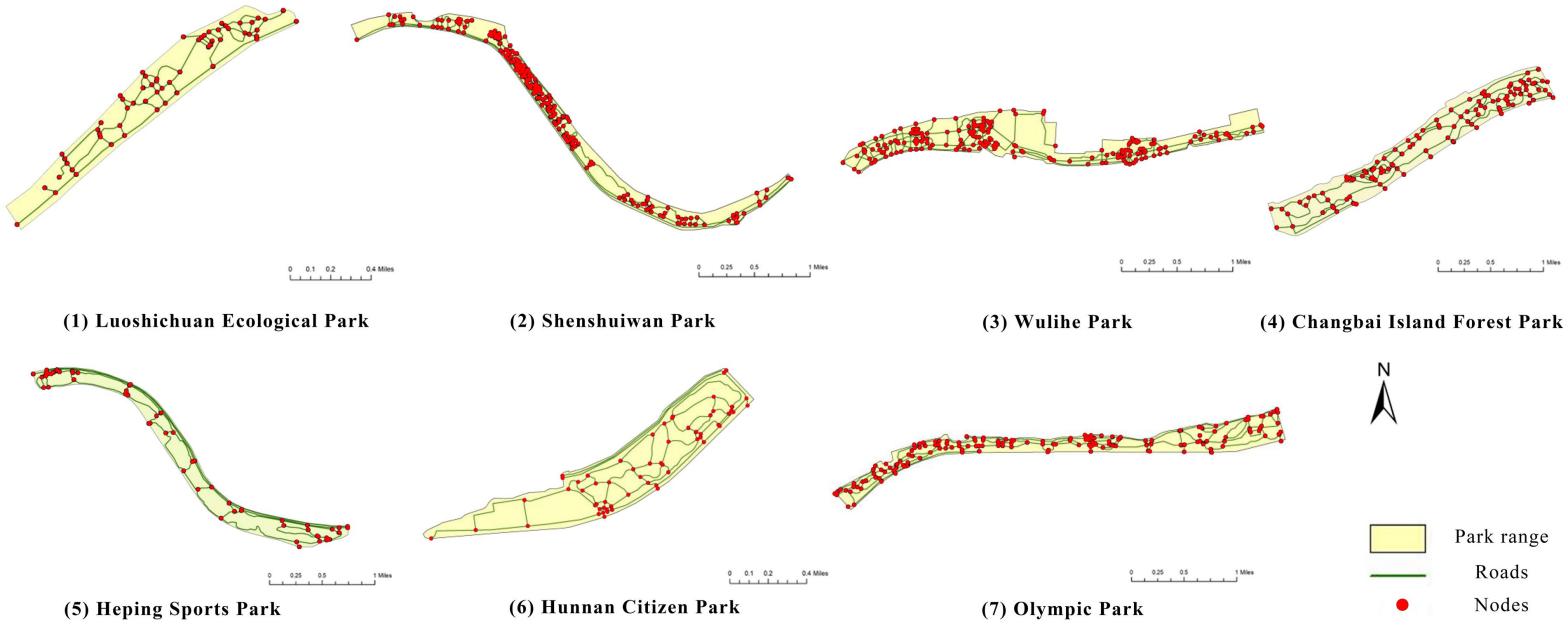
Road connectivity of each park.

**Figure 9:**
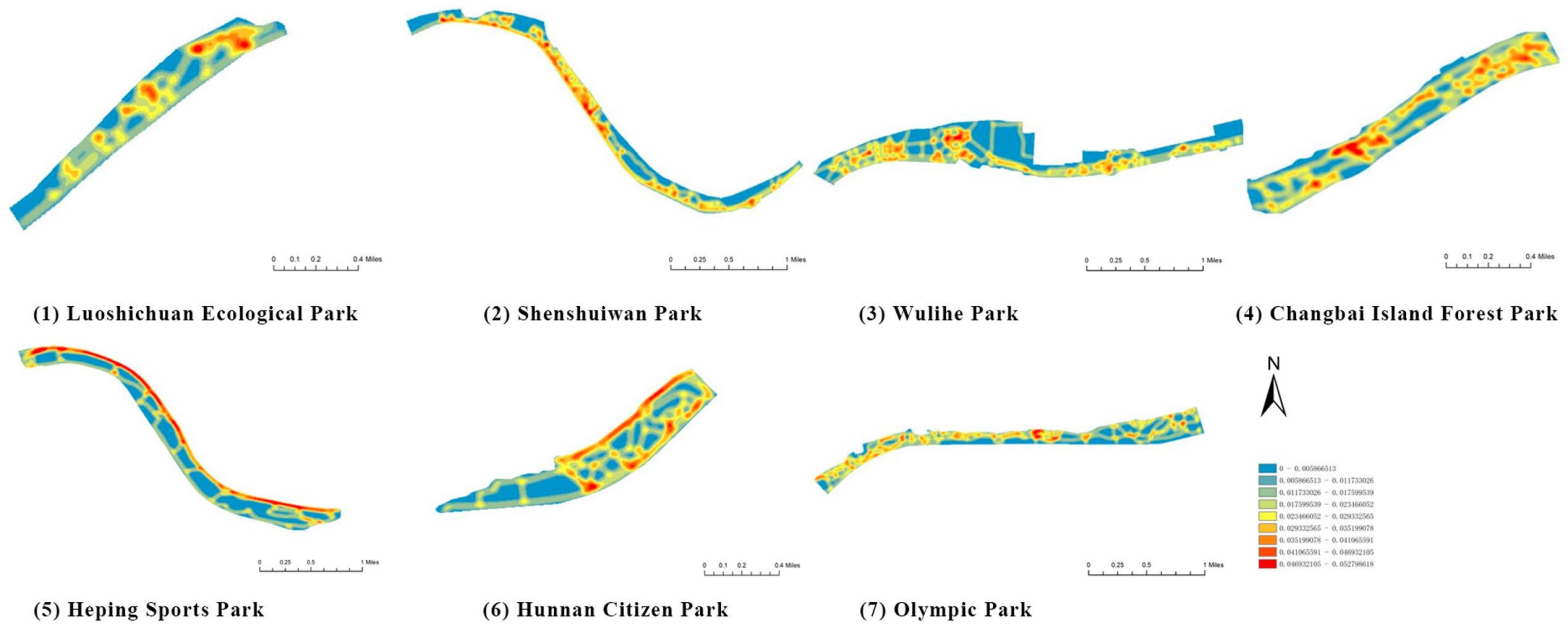
Slow-walking path density of each park.

**Figure 10:**
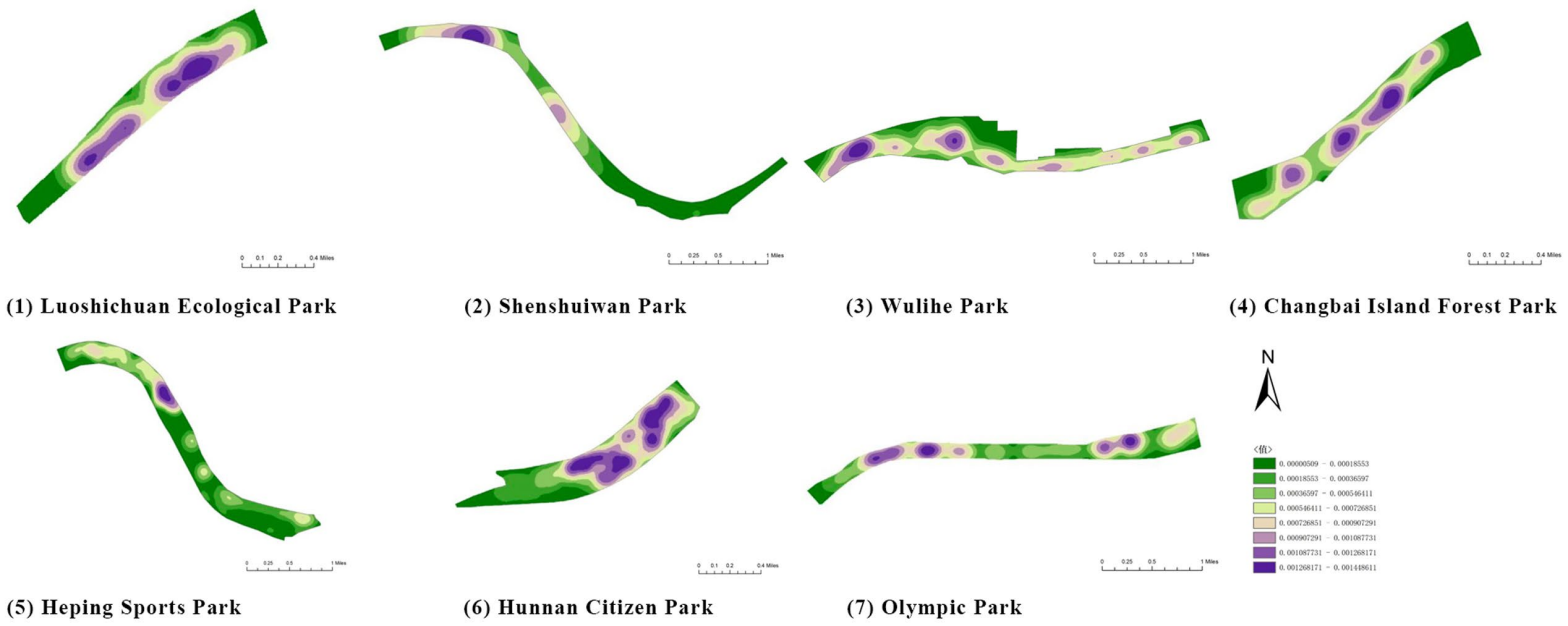
Visual chromaticity of each park.

Among the psychological health influence factors: Spatial safety perception (C1) and spatial privacy (C2) are quantified subjectively. The remaining five factors-spatial openness (C3), green view index (C4), road connectivity (C5), slow-walking path density (C6), and visual chromaticity (C7)-are quantified objectively. The acquisition and analysis of these objective indicators are as follows (Table 10):

(1) Spatial openness (Figure 6; Table 11): Calculated using the “Cat’s Eye Quadrant” image segmentation tool, consistent with the method for green view index. Results: Heping Sports Park (19.84%) and Hunnan Citizen Park (18.47%) had relatively high sky openness, with maximum values reaching 62.91 and 65.82%. This is because both parks have grassland areas enclosed by trees. Shenshuiwan Park had the lowest value (9.12%), with the north bank generally less open due to proximity to high-rise residential areas. Ranking: Heping Sports Park > Hunnan Citizen Park > Olympic Park > Changbai Island Forest Park > Luoshichuan Ecological Park > Wulihe Park > Shenshuiwan Park.
(2) Green view index (Figure 7; Table 12): On clear weekends in October 2022, photos were taken at 20–30 m intervals from the human perspective throughout each park. Data were extracted using the “Cat’s Eye Quadrant” tool and spatial distribution was modeled through ArcGIS kriging interpolation. Luoshichuan Ecological Park ranked highest, with max, min, and average values (48.46%) all leading, due to its ecological theme and rich vegetation. Heping Sports Park followed, while Changbai Island Forest Park and Wulihe Park scored below 40% due to large open activity spaces. Ranking: Luoshichuan Ecological Park > Heping Sports Park > Hunnan Citizen Park > Shenshuiwan Park > Olympic Park > Changbai Island Forest Park > Wulihe Park.
(3) Road connectivity (Figure 8; Table 13): Reflects the mesh structure and accessibility of internal park roads, calculated by extracting nodes and segments of road networks via GIS. Olympic Park had the highest connectivity (2.95), followed by Hunnan Citizen Park (2.90). Luoshichuan Ecological Park was lowest (2.52), suggesting more dead-end roads. Ranking: Olympic Park > Hunnan Citizen Park > Changbai Island Forest Park > Shenshuiwan Park > Heping Sports Park > Wulihe Park > Luoshichuan Ecological Park.
(4) Slow-walking path density (Figure 9; Table 14): Based on OpenStreetMap data supplemented by field surveys, calculated as road length per unit area. Shenshuiwan Park ranked highest (0.0219), followed by Olympic Park (0.0187). Heping Sports Park was lowest (0.0161). Parks with higher density (Shenshuiwan, Changbai Island Forest, and Olympic) generally had more woodland trails, while Hunnan Citizen Park and Heping Sports Park were fragmented by water bodies or large spaces, concentrating their paths. Ranking: Shenshuiwan Park > Changbai Island Forest Park > Olympic Park > Wulihe Park > Hunnan Citizen Park > Luoshichuan Ecological Park > Heping Sports Park.
(5) Visual chromaticity (Figure 10; Table 15): Using Toolsou to analyze the main hue values of survey photos. Higher values indicate cooler tones. Changbai Island Forest Park and Olympic Park had lower hue values (warmer tones). Luoshichuan Ecological Park, with high vegetation coverage, had the highest hue values (cooler tones). Ranking: Changbai Island Forest Park > Olympic Park > Shenshuiwan Park > Wulihe Park > Heping Sports Park > Hunnan Citizen Park > Luoshichuan Ecological Park. Notably, visual chromaticity showed a negative correlation with health restorativeness-cooler tones may reduce psychological recovery effects.

**Table 10:** Mental health impact indicators and their quantitative methods.

Factor class	Indicator number	Name of indicator	Is it objective
Mental health impact factor	C1	Sense of spatial security	×
	C2	Spatial privacy	×
	C3	Openness of view	√
	C4	Green view ratio	√
	C5	Road connectivity	√
	C6	Density of slow-moving road network	√
	C7	Visual colorfulness	√

**Table 11:** Quantitative results of the visual field width of each park.

Park name	Minimum value	Maximum value	Average number	Standard deviation
Luoshichuan Ecological Park	0.01	40.72	11.12	9.97
Shenshuiwan Park	0	34.67	9.12	8.94
Wulihe Park	0	45.76	10.79	9.25
Changbai Island Forest Park	0.04	41.88	11.15	8.85
Heping Sports Park	0.02	62.19	19.84	14.65
Hunnan Citizen Park	0	65.82	18.47	14.02
Olympic Park	0.01	56.44	17.73	13.80

**Table 12:** Quantitative results of green vision rate in each park.

Park name	Minimum value	Maximum value	Average number	Standard deviation
Luoshichuan Ecological Park	6.08	92.17	48.46	16.91
Shenshuiwan Park	0.32	87.56	43.57	19.60
Wulihe Park	1.89	80.01	38.11	15.85
Changbai Island Forest Park	1.4	74.68	39.12	16.08
Heping Sports Park	0.51	90.85	47.09	19.98
Hunnan Citizen Park	2.2	83.59	45.53	17.57
Olympic Park	3.09	91.54	41.88	17.81

**Table 13:** Road connectivity of each park.

Park name	Total number of road network sections	Total number of road network nodes	Road network connectivity
Luoshichuan Ecological Park	77	61	2.52
Shenshuiwan Park	325	229	2.85
Wulihe Park	363	197	2.65
Changbai Island Forest Park	169	117	2.89
Heping Sports Park	78	56	2.79
Hunnan Citizen Park	87	60	2.90
Olympic Park	232	160	2.97

**Table 14:** Quantitative results of slow-moving road network density in each park.

Park name	Road network length	Area area	Slow road network density
Luoshichuan Ecological Park	7017.19	401,363	0.0174
Shenshuiwan Park	26681.10	1,213,550	0.0219
Wulihe Park	301761.13	1,810,750	0.0186
Changbai Island Forest Park	15364.78	838,474	0.0188
Heping Sports Park	22790.17	1,332,030	0.0161
Hunnan Citizen Park	14871.08	806,254	0.0184
Olympic Park	26562.08	1,416,770	0.0187

**Table 15:** Quantitative results of visual color intensity for each park.

Park name	Minimum value	Maximum value	Average number	standard deviation
Luoshichuan Ecological Park	52	180	116.21	25.54
Shenshuiwan Park	30	133	89.43	22.36
Wulihe Park	36	138	92.77	21.28
Changbai Island Forest Park	28	121	70.11	24.19
Heping Sports Park	41	159	95.57	20.65
Hunnan Citizen Park	43	165	107.19	24.94
Olympic Park	35	124	71.62	25.18

##### 3.2.1.2 Physiological health influence factors

**Figure 11:**
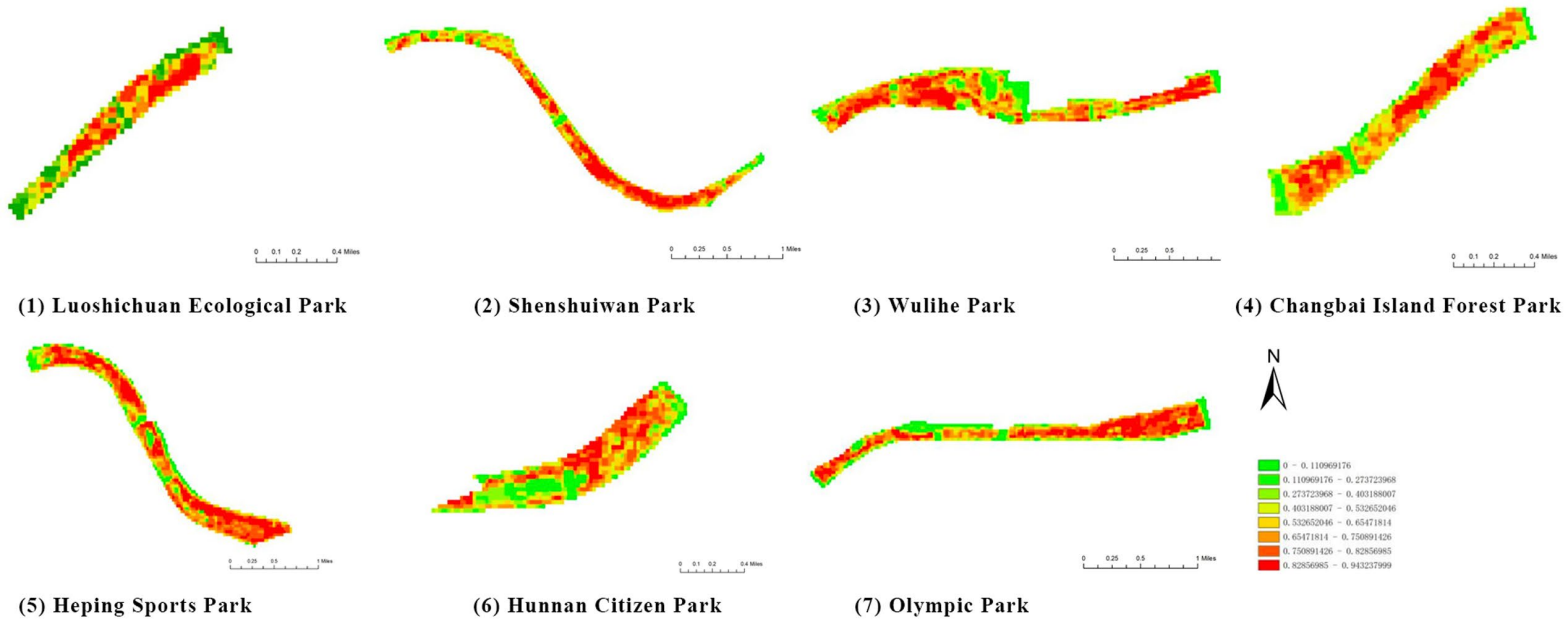
Vegetation coverage rate of each park.

Among the physiological health influence factors, vegetation type, vegetation coverage, and revetment type were quantified objectively, while environmental quietness, environmental sanitation, and waterfront landscape aesthetics were quantified subjectively. The objective indicators are analyzed as follows (Table 16):

(1) Vegetation type (Table 17): The riverside parks along the Hunhe River feature a rich diversity of plant types. Most parks are composed of a combination of trees, shrubs, and turf, though some parks exhibit certain differences in plant variety. Detailed field surveys of vegetation types in each park revealed a certain regularity in their distribution. Overall, tree species dominate in most parks, while shrubs and turf act as supplementary vegetation in different areas, together forming a rich plant hierarchy.
(2) Vegetation coverage rate (Figure 11; Table 18): Vegetation coverage was calculated using the NDVI (Normalized Difference Vegetation Index) in ENVI software, with results converted into raster data for processing and analysis in GIS. Luoshichuan Ecological Park had the highest vegetation coverage among the seven parks, with an average of 78.62%, largely due to fewer activity facilities and extensive green spaces. Changbai Island Forest Park followed at 75.18%, with its layered landscape and internal water systems significantly contributing to vegetation coverage. Shenshuiwan Park had the lowest coverage, largely constrained by buildings, activity fields, and plazas, as well as road and cycling path arrangements that occupy space otherwise suitable for greenery. Ranking (high to low): Luoshichuan Ecological Park > Changbai Island Forest Park > Olympic Park > Heping Sports Park > Wulihe Park > Hunnan Citizen Park > Shenshuiwan Park.
(3) Embankment type (Table 19): The riverbank types of the seven parks were investigated and categorized into natural and artificial, with artificial revetments further divided into stepped and vertical types. Results showed that natural and vertical revetments are most common in the Hunhe riverside parks, though their distribution varies among parks. Heping Sports Park and Hunnan Citizen Park contained only one type of revetment, while the others featured two or more. Notably, Olympic Park exhibited the highest diversity, incorporating natural, vertical, and stepped revetments. Field observations indicated that natural revetments had the highest usage rate, typically for fishing or water play. Vertical revetments, on the other hand, had relatively low usage due to aging facilities, with unsatisfactory conditions in all parks except Shenshuiwan Park. Comprehensive ranking of revetment type scores (high to low): Olympic Park > Wulihe Park = Changbai Island Forest Park > Hunnan Citizen Park > Luoshichuan Ecological Park = Shenshuiwan Park > Heping Sports Park.

**Table 16:** Physiological health impact indicators and their quantitative methods.

Factor class	Indicator number	Name of indicator	Is it objective
Mental health impact factor	C8	Vegetation type	√
	C9	Vegetation coverage rate	√
	C10	Environmental quietness	×
	C11	Environmental hygiene	×
	C12	Embankment type	√
	C13	Water Landscape Aesthetics	×

**Table 17:** Vegetation types of each park.

Park name	Vegetation type	Type	Score
Luoshichuan Ecological Park	Trees, Shrubs, Turf	3	3
Shenshuiwan Park	Trees, Shrubs, Turf, flowers	4	5
Wulihe Park	Trees, Shrubs, Tur	3	3
Changbai Island Forest Park	Trees, Shrubs, Turf, flowers	3	5
Heping Sports Park	Trees, Turf	2	2
Hunnan Citizen Park	Trees, Turf	2	2
Olympic Park	Trees, Shrubs, Tur	3	3

**Table 18:** Quantitative results of vegetation coverage in each park.

Park name	Minimum value	Maximum value	Average number	standard deviation
Luoshichuan Ecological Park	52	180	116.21	25.54
Shenshuiwan Park	30	133	89.43	22.36
Wulihe Park	36	138	92.77	21.28
Changbai Island Forest Park	28	121	70.11	24.19
Heping Sports Park	41	159	95.57	20.65
Hunnan Citizen Park	43	165	107.19	24.94
Olympic Park	35	124	71.62	25.18

**Table 19:** Types of revetment in each park and quantitative results of various park revetment types.

Park name	Natural revetment	Artificial vertical revetment	Artificial stepped revetment	Score
Luoshichuan Ecological Park	×	√	√	2
Shenshuiwan Park	×	√	√	2
Wulihe Park	√	×	√	3
Changbai Island Forest Park	√	×	√	3
Heping Sports Park	×	×	√	0.5
Hunnan Citizen Park	√	×	×	2.5
Olympic Park	√	×	√	3

##### 3.2.1.3 Social interaction influence factors

**Figure 12:**
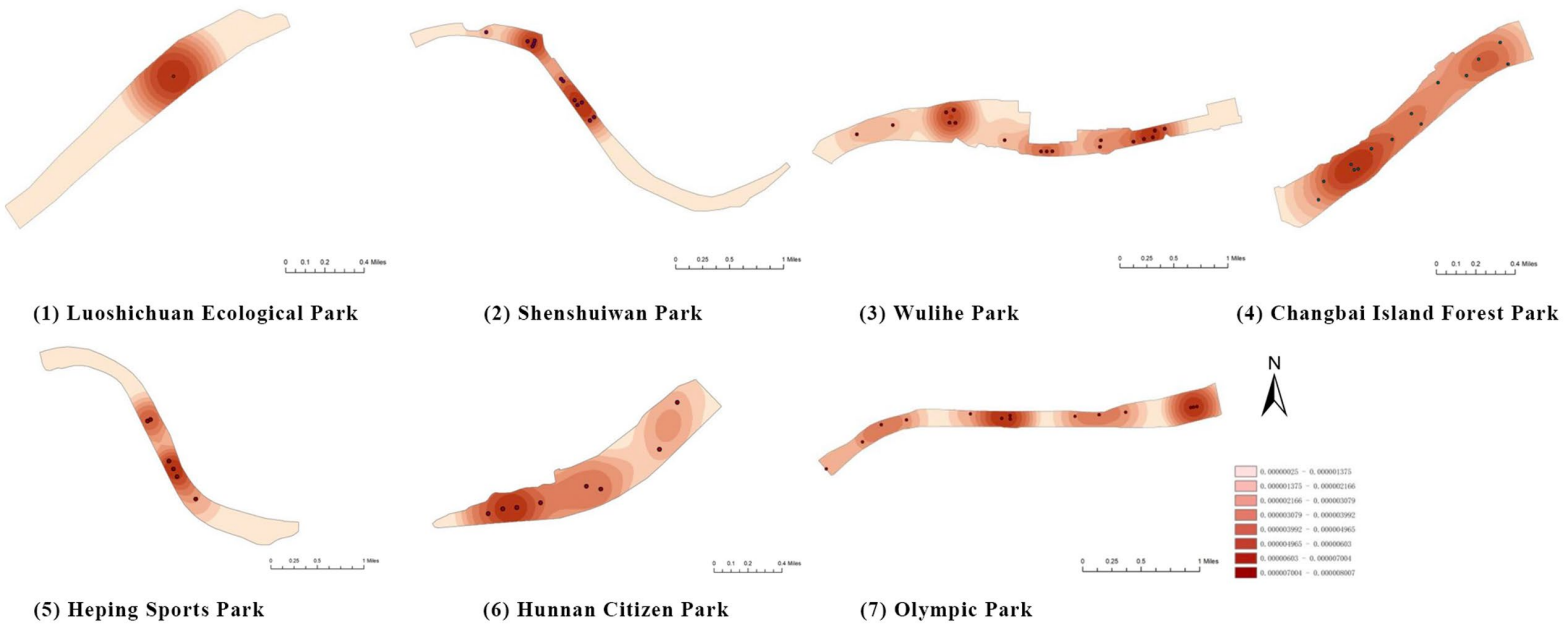
Number of recreational and fitness facilities in each park.

**Figure 13:**
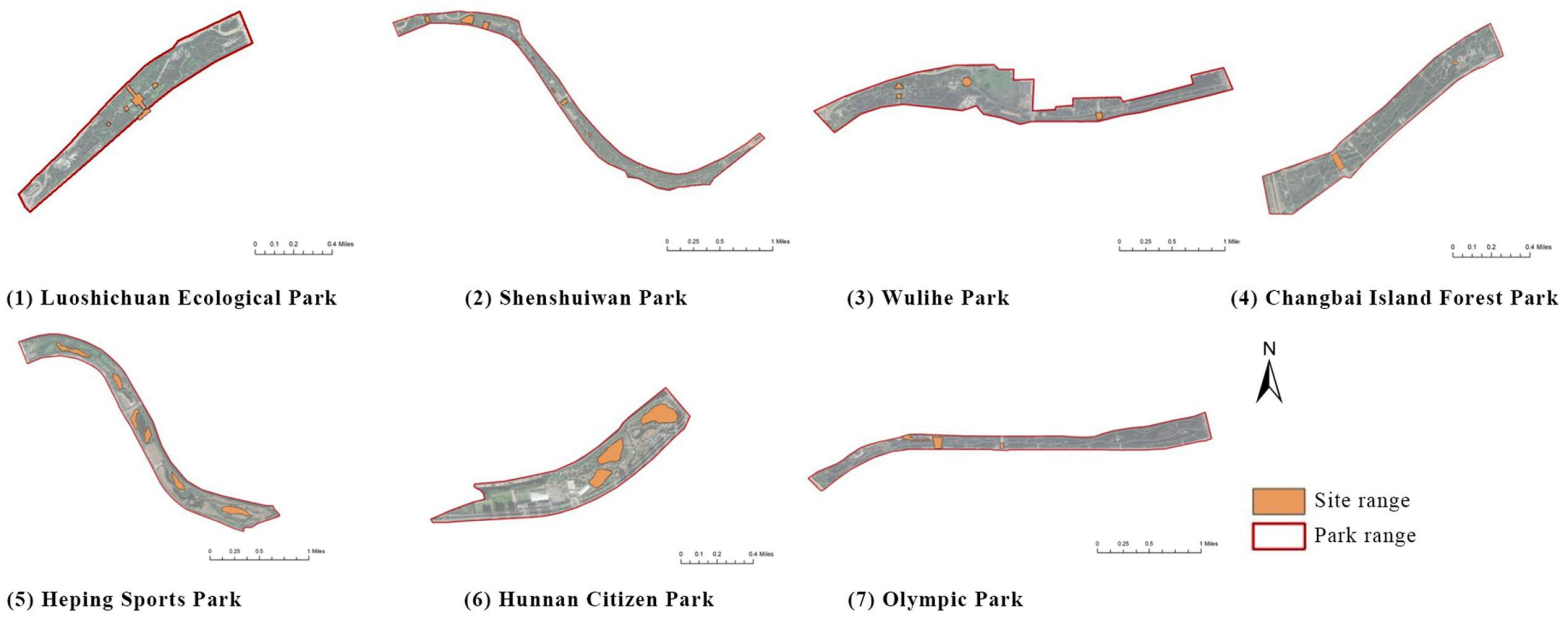
Activity space area of each park.

**Figure 14:**
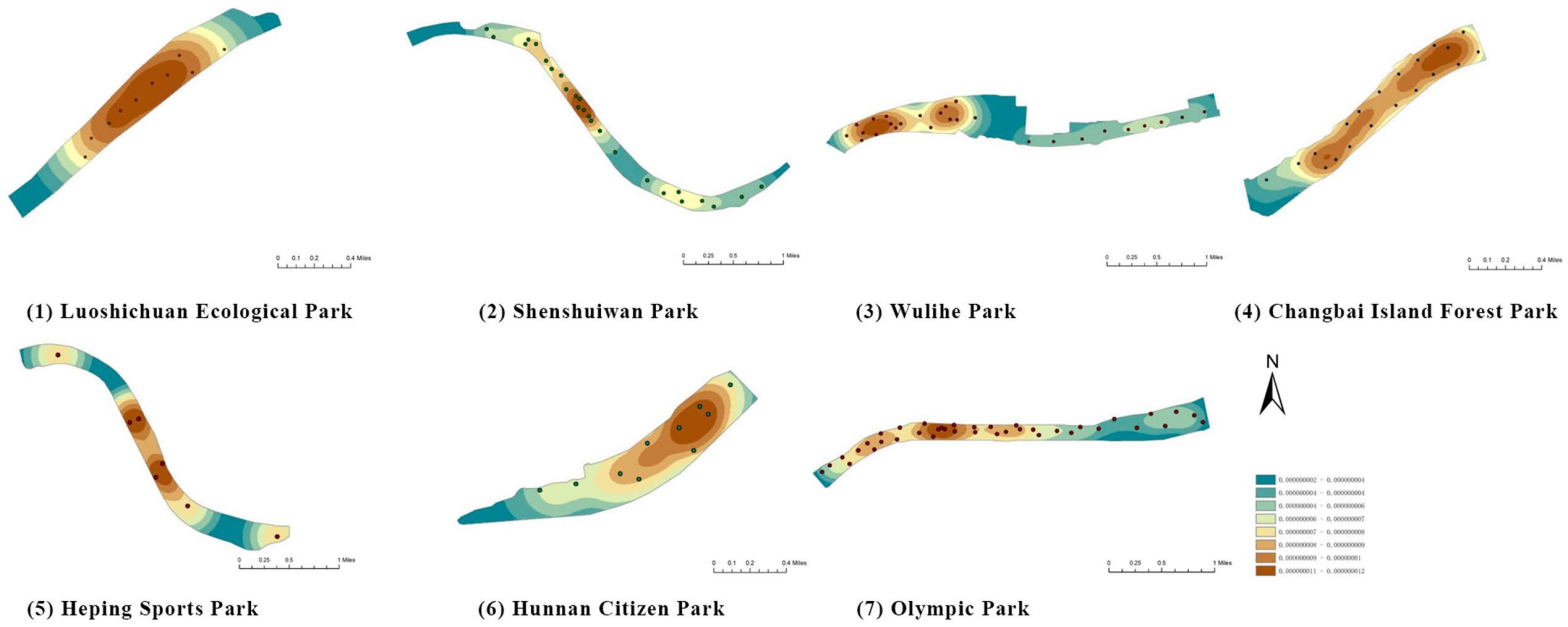
Number of rest facilities of each park.

Among the social interaction health influence factors, types of recreational and fitness facilities, number of recreational and fitness facilities, and activity space area were quantified objectively, while the number of rest facilities was quantified subjectively. The objective indicators are analyzed as follows (Table 20):

(1) Variety of recreational and fitness facilities (Table 21): The diversity of recreational and fitness facilities directly affects the ability to meet the increasingly diverse fitness needs of residents. Field surveys recorded facility types such as fitness equipment, soccer fields, basketball courts, badminton courts, table tennis courts, tennis courts, playgrounds, and skateparks, with each facility type assigned a score of 1. Wulihe Park ranked the highest, offering nearly all major ball game facilities as well as a newly added skatepark that caters to specific user groups. Shenshuiwan Park and Olympic Park followed, each with five types of facilities. Heping Sports Park had only a soccer field, while Luoshichuan Ecological Park contained no recreational or fitness facilities. Ranking (high to low): Wulihe Park > Shenshuiwan Park = Olympic Park > Changbai Island Forest Park > Hunnan Citizen Park > Heping Sports Park > Luoshichuan Ecological Park.
(2) Number of recreational and fitness facilities (Figure 12; Table 22): The quantity and spatial distribution of facilities reflect the extent to which parks satisfy residents’ fitness demands. Field surveys combined with kernel density analysis in ArcGIS were used to assess this. Wulihe Park had the largest number of facilities (15), evenly distributed across the park. Olympic Park followed with 14 facilities, broadly covering the park. Shenshuiwan Park had 11 facilities, but with more clustered distributions, featuring multiple facility types within the same areas. Changbai Island Forest Park and Heping Sports Park had relatively concentrated facilities located in specific areas, with a limited variety. Luoshichuan Ecological Park had only one set of fitness equipment. Ranking (high to low): Wulihe Park > Olympic Park > Shenshuiwan Park > Changbai Island Forest Park > Hunnan Citizen Park > Heping Sports Park > Luoshichuan Ecological Park.
(3) Activity space area (Figure 13; Table 23): The size of activity spaces directly influences the diversity and freedom of park activities. Based on current condition surveys and ArcGIS statistical analysis, the area and number of activity spaces in each park were calculated. Heping Sports Park and Hunnan Citizen Park had the largest activity spaces, mostly open grass fields suitable for diverse activities such as kite-flying, skateboarding, and group dancing, with high spatial openness and broad visibility. Shenshuiwan Park had the largest number of activity spaces, evenly distributed, allowing easy access from multiple entrances. Wulihe Park had moderately sized spaces, more concentrated in specific areas. Ranking (high to low): Heping Sports Park > Hunnan Citizen Park > Shenshuiwan Park > Olympic Park > Wulihe Park > Luoshichuan Ecological Park > Changbai Island Forest Park.
(4) Number of rest facilities (Figure 14; Table 24): The number and distribution of rest facilities reflect the park’s capacity to meet users’ needs for rest. Field surveys identified the locations of rest facilities, and kernel density analysis in ArcGIS was used to evaluate distribution patterns. Olympic Park had the largest number of rest facilities (37), with a diverse range including waterside benches and forest seating. Wulihe Park ranked second, with facilities mainly located along the main roads. Shenshuiwan Park, Changbai Island Forest Park, and Luoshichuan Ecological Park had relatively fewer rest facilities. Heping Sports Park had the fewest, where most visitors had to sit directly on the ground. Ranking (high to low): Olympic Park > Wulihe Park > Shenshuiwan Park > Changbai Island Forest Park > Luoshichuan Ecological Park > Hunnan Citizen Park > Heping Sports Park.

**Table 20:** Social interaction health impact indicators and their quantitative methods.

Factor calass	Indicator number	Name of indicator	Is it objective
Mental health impact factor	C14	Variety of Recreational and Fitness Facilities	√
	C15	Number of Recreational and Fitness Facilities	√
	C16	Number of Scenic Facilities	×
	C17	Quality of Scenic Facilities	×
	C18	Activity Area Size	√
	C19	Number of Resting Facilities	√

**Table 21:** Indicators and quantification methods for the impact of social interaction on health.

Park name	Callisthenic apparatus	Football field	Basketball court	Badminton field	Table tennis court	tennis court	Playground	Skateboard field	Score
Luoshichuan Ecological Park									0
Shenshuiwan Park	√	√	√		√	√			5
Wulihe Park	√	√	√		√	√		√	6
Changbai Island Forest Park		√	√		√		√		4
Heping Sports Park		√							1
Hunnan Citizen Park	√	√		√		√			4
Olympic Park	√	√	√	√		√			5

**Table 22:** Quantitative results of the number of entertainment and fitness facilities in each park.

Park name	Quantity	Area
Luoshichuan Ecological Park	1	324
Shenshuiwan Park	11	21,644
Wulihe Park	15	26,513
Changbai Island Forest Park	9	6,471
Heping Sports Park	5	1,329
Hunnan Citizen Park	8	24,632
Olympic Park	14	29,157

**Table 23:** Quantitative results of activity area in each park.

Park name	Activity area of the site	Park area	Number of sites
Luoshichuan Ecological Park	14,570	401,363	5
Shenshuiwan Park	42,919	1,213,550	8
Wulihe Park	31,708	1,810,750	4
Changbai Island Forest Park	11,945	838,474	2
Heping Sports Park	123,934	1,332,030	6
Hunnan Citizen Park	98,630	806,254	3
Olympic Park	42,212	1,416,770	3

**Table 24:** The number of rest facilities in each park.

Park name	Quantity
Luoshichuan Ecological Park	11
Shenshuiwan Park	24
Wulihe Park	27
Changbai Island Forest Park	19
Heping Sports Park	7
Hunnan Citizen Park	10
Olympic Park	37

#### 3.2.2 Comprehensive evaluation results analysis

##### 3.2.2.1 Overall evaluation results

This section provides a comprehensive evaluation of the health restorative capacity of the seven studied parks. First, the evaluation results of the psychological health, physiological health, and social interaction health influence factors for each park were dimensionless-processed, with the sum-normalization method applied to unify the scale of each indicator. Weighted calculations were then performed according to the weights assigned to each park. The normalized results are presented in Table 25.

**Table 25:** Normalized weighted results for each indicator.

Indicator number	Name of indicator	Luoshichuan Ecological Park	Shenshuiwan Park	Wulihe Park	Changbai Island Forest Park	Heping Sports Park	Hunnan Citizen Park	Olympic Park
C1	Sense of spatial security	0.14	0.15	0.16	0.15	0.14	0.14	0.13
C2	Spatial privacy	0.14	0.17	0.15	0.14	0.14	0.13	0.15
C3	Openness of view	0.11	0.09	0.11	0.11	0.20	0.19	0.18
C4	Green view ratio	0.16	0.14	0.13	0.13	0.16	0.15	0.14
C5	Road connectivity	0.13	0.15	0.14	0.15	0.14	0.15	0.15
C6	Density of slow-moving road network	0.13	0.17	0.14	0.14	0.12	0.14	0.14
C7	Visual colorfulness	0.18	0.14	0.14	0.11	0.15	0.17	0.11
C8	Vegetation type	0.13	0.22	0.11	0.22	0.09	0.09	0.13
C9	Vegetation coverage rate	0.16	0.12	0.22	0.18	0.15	0.12	0.15
C10	Environmental quietness	0.14	0.14	0.18	0.13	0.16	0.16	0.13
C11	Environmental hygiene	0.16	0.14	0.13	0.13	0.15	0.14	0.15
C12	Embankment type	0.13	0.13	0.19	0.19	0.03	0.16	0.19
C13	Water landscape aesthetics	0.11	0.15	0.15	0.15	0.15	0.15	0.15
C14	Variety of recreational and fitness facilities	0.03	0.19	0.23	0.15	0.04	0.15	0.19
C15	Number of recreational and fitness facilities	0.03	0.27	0.21	0.12	0.00	0.06	0.30
C16	Number of scenic facilities	0.15	0.20	0.20	0.10	0.10	0.05	0.20
C17	Quality of scenic facilities	0.10	0.20	0.15	0.10	0.15	0.05	0.25
C18	Activity area size	0.08	0.13	0.12	0.09	0.23	0.20	0.15
C19	Number of resting facilities	0.137	0.205	0.192	0.103	0.055	0.068	0.240

**Figure 15:**
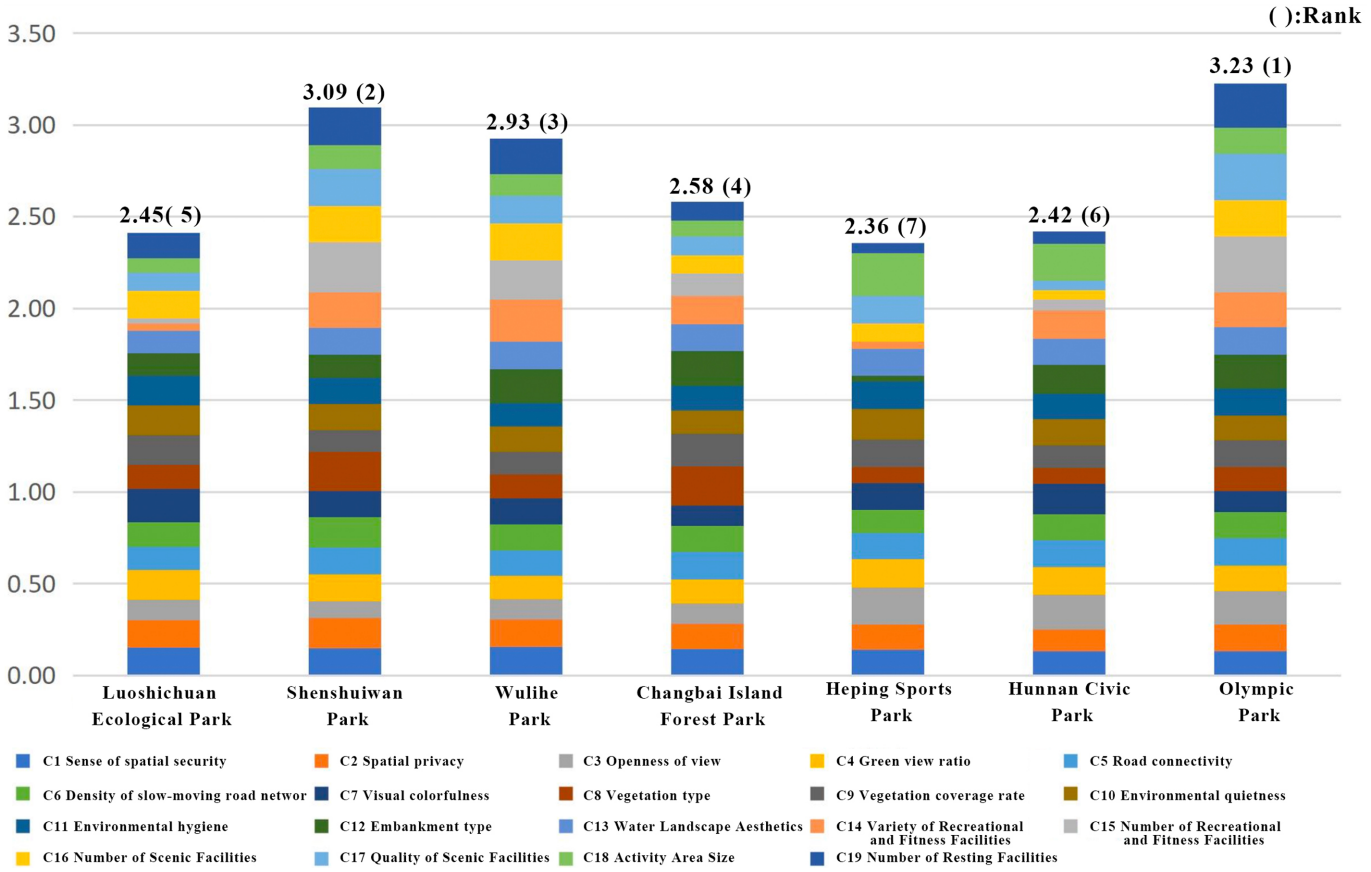
Health recovery scores and ranking of each park.

By summing the weighted scores of each indicator, the final Health Restoration Index of each park was obtained (Figure 15). This index reflects the degree to which each park contributes to the health restoration of urban residents: a higher score indicates stronger restorative effectiveness, while a lower score indicates weaker effectiveness. According to the results, Olympic Park achieved the highest Health Restoration Index at 3.23, followed by Shenshuiwan Park (3.09) and Wulihe Park (2.93). Heping Sports Park scored the lowest, with an index of 2.36. The ranking of Health Restoration Index values reveals significant differences in the restorative effectiveness of the parks.

Further analysis (Table 26) of the scores in mental health, physiological health, and social interaction health factors indicates that Olympic Park performed best in psychological health and social interaction health, whereas Changbai Island Forest Park scored higher in physiological health. In particular, Olympic Park’s score for social interaction health factors ranked first, reflecting the park’s strong role in promoting resident social interactions. Although the overall health restorative scores varied across the parks, each park demonstrated different dominant indicators; therefore, a single factor ranking cannot fully capture the effectiveness of health restoration. The next step will further analyze the dominant indicators of each park to identify key factors contributing most to health restoration, providing guidance for future park planning and optimization.

**Table 26:** Evaluation results of each factor class in each park.

Factor calss	Evaluation criteria	Luoshichuan Ecological Park	Shenshuiwan Park	Wulihe Park	Changbai Island Forest Park	Heping Sports Park	Hunnan Citizen Park	Olympic Park
Mental health impact factor	Score	0.80	0.86	0.83	0.79	0.90	0.90	0.92
	Rank	6	4	5	7	2	3	1
Physiological health impact factor	Score	0.89	0.86	0.84	0.99	0.73	0.79	0.89
	Rank	2	4	5	1	7	6	3
Social interaction impact factor	Score	0.53	1.20	1.11	0.67	0.58	0.59	1.33
	Rank	7	2	3	4	6	5	1

##### 3.2.2.2 Evaluation results of each park

(1) Luoshichuan Ecological Park: The analysis (Table 27) shows that for Luoshichuan Ecological Park, physiological health influence factors contributed the most to its Health Restoration Index, serving as the dominant factor in its restorative function. By contrast, social interaction influence factors performed weakly, becoming the park’s disadvantage. Specifically: In psychological health, the dominant advantage was C4: vegetation coverage green view index. In physiological health, the main advantages were C9: vegetation coverage rate, C10: environmental quietness, and C11: environmental sanitation. In social interaction health, the overall performance was relatively average.

**Table 27:** Scores and ranking of each factor of Luoshichuan Ecological Park.

Factor class		Index	Index score	Rank
Mental health impact factor	C1	Sense of spatial security	0.111	7
	C2	Spatial privacy	0.112	7
	C3	Openness of view	0.113	5
	**C4**	**Green view ratio**	**0.161**	**1**
	C5	Road connectivity	0.129	7
	C6	Density of slow-moving road network	0.134	6
	**C7**	**Visual colorfulness**	**0.181**	**1**
Physiological health impact factor	C8	Vegetation type	0.130	3
	**C9**	**Vegetation coverage rate**	**0.163**	**2**
	**C10**	**Environmental quietness**	**0.175**	**1**
	**C11**	**Environmental hygiene**	**0.180**	**1**
	C12	Embankment type	0.125	5
	C13	Water landscape aesthetics	0.121	7
Mental health impact factor	C14	Variety of recreational and fitness facilities	0.038	6
	C15	Number of recreational and fitness facilities	0.031	6
	C16	Number of scenic facilities	0.150	5
	C17	Quality of scenic facilities	0.105	5
	C18	Activity area size	0.077	7
	C19	Number of resting facilities	0.137	4

*The bolded values in the table indicates that the factor’s score and ranking are significant.

Overall, Luoshichuan Ecological Park ranked fifth among the seven parks in health restorative effectiveness. Physiological health factors played the leading role, with psychological health factors serving as auxiliary contributors, while social interaction factors represented the main area for improvement. Therefore, in future enhancements of restorative capacity, priority should be given to reinforcing the physiological health restorative effects.

(2) Shenshuiwan Park: The analysis (Table 28) shows that for Shenshuiwan Park, social interaction health influence factors contributed the most to its Health Restoration Index, serving as the dominant factor. Psychological health factors performed relatively weakly, becoming the park’s disadvantage. In terms of specific indicators: Psychological health: The advantage lies in C6: Density of Slow-Traffic Network. Physiological health: The advantages include C8: Vegetation Diversity and C13: Water Landscape Attractiveness. Social interaction health: Multiple indicators were advantageous, including C14: Variety of Fitness Facilities, C15: Number of Fitness Facilities, C16: Number of Recreational Facilities, and C19: Number of Rest Facilities.

**Table 28:** Scores and ranking of each factor of Shenshuiwan Park.

Factor class		Index	Index score	Rank
Mental health impact factor	C1	Sense of spatial security	0.148	3
	C2	Spatial privacy	0.165	2
	C3	Openness of view	0.093	7
	C4	Green view ratio	0.143	4
	C5	Road connectivity	0.146	4
	**C6**	**Density of slow-moving road network**	**0.169**	**1**
	C7	Visual colorfulness	0.139	5
Physiological health impact factor	**C8**	**Vegetation type**	**0.217**	**1**
	C9	Vegetation coverage rate	0.116	7
	C10	Environmental quietness	0.123	7
	C11	Environmental hygiene	0.132	6
	C12	Embankment type	0.125	5
	**C13**	**Water landscape aesthetics**	**0.148**	**1**
Mental health impact factor	**C14**	**Variety of recreational and fitness facilities**	**0.192**	**2**
	**C15**	**Number of recreational and fitness facilities**	**0.273**	**2**
	**C16**	**Number of scenic facilities**	**0.214**	**1**
	**C17**	**Quality of scenic facilities**	**0.221**	**2**
	C18	Activity area size	0.133	4
	**C19**	**Number of resting facilities**	**0.205**	**2**

*The bolded values in the table indicates that the factor’s score and ranking are significant.

Overall, Shenshuiwan Park ranked second among the seven parks in restorative effectiveness. Social interaction factors dominated, while psychological and physiological factors played relatively balanced supporting roles. Future improvements should further strengthen the social interaction functions, maximizing the use of existing resources to enhance restorative effectiveness.

(3) Wulihe Park: For Wulihe Park (Table 29), social interaction health factors also contributed the most, serving as the dominant factor in its restorative function. Specific advantages include: Psychological health: C1: sense of spatial safety. Physiological health: C12: bank type and C13: water landscape attractiveness. Social interaction health: C14: variety of fitness facilities and C16: number of recreational facilities.

**Table 29:** Scores and ranking of each factor of Wulihe Park.

Factor class		Index	Index score	Rank
Mental health impact factor	**C1**	**Sense of spatial security**	**0.157**	**1**
	C2	Spatial privacy	0.151	3
	C3	Openness of view	0.112	6
	C4	Green view ratio	0.126	7
	C5	Road connectivity	0.136	6
	C6	Density of slow-moving road network	0.143	4
	C7	Visual colorfulness	0.144	4
Physiological health impact factor	C8	Vegetation type	0.130	3
	C9	Vegetation coverage rate	0.124	5
	C10	Environmental quietness	0.136	4
	C11	Environmental hygiene	0.116	7
	**C12**	**Embankment type**	**0.188**	**1**
	**C13**	**Water landscape aesthetics**	**0.148**	**1**
Mental health impact factor	**C14**	**Variety of recreational and fitness facilities**	**0.231**	**1**
	C15	Number of recreational and fitness facilities	0.212	3
	**C16**	**Number of scenic facilities**	**0.213**	**1**
	C17	Quality of scenic facilities	0.150	3
	C18	Activity area size	0.122	5
	C19	Number of resting facilities	0.192	3

*The bolded values in the table indicates that the factor’s score and ranking are significant.

Overall, Wulihe Park ranked third in health restorative effectiveness, with outstanding performance. Social interaction health factors dominated, while psychological and physiological factors played auxiliary roles. Future efforts should focus on strengthening social interaction functions and leveraging existing facilities to further enhance restorative effects.

(4) Changbai Island Forest Park: For Changbai Island Forest Park (Table 30), physiological health influence factors contributed the most, serving as the dominant factor. Psychological and social interaction health factors provided relatively balanced contributions. Specifically: psychological health: C5: road connectivity and C6: density of slow-traffic network were advantages. Physiological health: C8: vegetation diversity, C9: vegetation coverage rate, C12: bank type, and C13: water landscape attractiveness were advantages. Social interaction health: Overall performance was average.

**Table 30:** Scores and ranking of each factor of Changbai Island Forest Park.

Factor class		Index	Index score	Rank
Mental health impact factor	C1	Sense of spatial security	0.145	4
	C2	Spatial privacy	0.135	5
	C3	Openness of view	0.114	4
	C4	Green view ratio	0.129	6
	**C5**	**Road connectivity**	**0.148**	**2**
	**C6**	**Density of slow-moving road network**	**0.144**	**2**
	C7	Visual colorfulness	0.109	7
Physiological health impact factor	**C8**	**Vegetation type**	**0.217**	**1**
	**C9**	**Vegetation coverage rate**	**0.177**	**1**
	C10	Environmental quietness	0.127	6
	C11	Environmental hygiene	0.134	5
	**C12**	**Embankment type**	**0.188**	**1**
	**C13**	**Water landscape aesthetics**	**0.148**	**1**
Mental health impact factor	C14	Variety of recreational and fitness facilities	0.154	4
	C15	Number of recreational and fitness facilities	0.121	4
	C16	Number of scenic facilities	0.116	5
	C17	Quality of scenic facilities	0.112	5
	C18	Activity area size	0.090	6
	C19	Number of resting facilities	0.103	5

*The bolded values in the table indicates that the factor’s score and ranking are significant.

Overall, Changbai Island Forest Park ranked fourth in restorative effectiveness, with physiological health factors playing the dominant role, supported by psychological and social interaction factors. Future improvements should focus on strengthening natural attributes related to physiological health.

(5) Heping Sports Park: In Heping Sports Park (Table 31), psychological health influence factors contributed the most, serving as the dominant factor. Physiological and social interaction factors contributed relatively evenly, though activity facilities significantly restricted restorative effects. Specific advantages were: psychological health: C3: visual openness and C4: green view index. Physiological health: C10: environmental quietness, C11: environmental sanitation, and C13: water landscape attractiveness. Social interaction health: C18: area of activity space.

**Table 31:** Scores and ranking of each factor of Heping Sports Park.

Factor class		Index	Index score	Rank
Mental health impact factor	C1	Sense of spatial security	0.143	5
	C2	Spatial privacy	0.137	4
	**C3**	**Openness of view**	**0.202**	**1**
	**C4**	**Green view ratio**	**0.155**	**2**
	C5	Road connectivity	0.143	5
	C6	Density of slow-moving road network	0.124	7
	C7	Visual colorfulness	0.148	3
Physiological health impact factor	C8	Vegetation type	0.087	6
	C9	Vegetation coverage rate	0.151	3
	**C10**	**Environmental quietness**	**0.163**	**2**
	**C11**	**Environmental hygiene**	**0.152**	**2**
	C12	Embankment type	0.031	7
	**C13**	**Water landscape aesthetics**	**0.148**	**1**
Mental health impact factor	C14	Variety of recreational and fitness facilities	0.038	6
	C15	Number of recreational and fitness facilities	0.045	7
	C16	Number of scenic facilities	0.121	5
	C17	Quality of scenic facilities	0.152	3
	**C18**	**Activity area size**	**0.232**	**1**
	C19	Number of resting facilities	0.055	7

*The bolded values in the table indicates that the factor’s score and ranking are significant.

Overall, Heping Sports Park ranked sixth among the seven parks in restorative effectiveness. Psychological health factors dominated, physiological health factors played a supporting role, while social interaction factors remained the weakest link. Future improvements should focus on enhancing psychological restorative effects while compensating for deficiencies in social interaction.

(6) Hunnan Citizen Park: For Hunnan Citizen Park (Table 32), psychological health influence factors contributed the most, followed by physiological factors, while social interaction factors contributed the least. Specific advantages include: Psychological health: C3: visual openness, C5: road connectivity, and C7: visual colorfulness. Physiological health: Overall performance was average. Social interaction health: C18: area of activity space.

**Table 32:** Scores and ranking of each factor of Hunnan Citizen Park.

Factor class		Index	Index score	Rank
Mental health impact factor	C1	Sense of spatial security	0.131	6
	C2	Spatial privacy	0.122	6
	**C3**	**Openness of view**	**0.188**	**2**
	C4	Green view ratio	0.150	3
	**C5**	**Road connectivity**	**0.148**	**2**
	C6	Density of slow-moving road network	0.142	5
	**C7**	**Visual colorfulness**	**0.167**	**2**
Physiological health impact factor	C8	Vegetation type	0.087	6
	C9	Vegetation coverage rate	0.122	6
	C10	Environmental quietness	0.141	3
	C11	Environmental hygiene	0.141	4
	C12	Embankment type	0.156	4
	C13	Water landscape aesthetics	0.142	6
Mental health impact factor	C14	Variety of recreational and fitness facilities	0.154	4
	C15	Number of recreational and fitness facilities	0.061	5
	C16	Number of scenic facilities	0.051	7
	C17	Quality of scenic facilities	0.054	7
	**C18**	**Activity area size**	**0.204**	**2**
	C19	Number of resting facilities	0.068	6

*The bolded values in the table indicates that the factor’s score and ranking are significant.

Overall, Hunnan Citizen Park ranked seventh in restorative effectiveness, with generally modest outcomes. Psychological health factors dominated, supported by physiological health, while social interaction health factors constrained the overall effect. Future improvements should prioritize social interaction health factors by optimizing related facilities to enhance restorative capacity.

(7) Olympic Park: For Olympic Park (Table 33), social interaction health influence factors contributed the most, serving as the dominant factor in restorative capacity. Although the contributions of physiological and psychological health factors were slightly lower, their performance across all indicators was strong, with no obvious weaknesses. Specifically: Psychological health: Advantages include C1: sense of spatial safety, C2: spatial privacy, C5: road connectivity, and C6: density of slow-traffic network. Physiological health: C12: bank type and C13: water landscape Attractiveness were advantages. Social interaction health: Multiple indicators showed advantages, including C14: variety of fitness facilities, C15: number of fitness facilities, C16: number of recreational facilities, C17: quality of recreational facilities, and C19: number of rest facilities.

**Table 33:** Scores and ranking of each factor of Olympic Park.

Factor class		Index	Index score	Rank
Mental health impact factor	**C1**	**Sense of spatial security**	**0.152**	**2**
	**C2**	**Spatial privacy**	**0.196**	**1**
	C3	Openness of view	0.181	3
	C4	Green view ratio	0.138	5
	**C5**	**Road connectivity**	**0.151**	**1**
	**C6**	**Density of slow-moving road network**	**0.144**	**2**
	C7	Visual colorfulness	0.113	6
Physiological health impact factor	C8	Vegetation type	0.131	3
	C9	Vegetation coverage rate	0.147	4
	C10	Environmental quietness	0.133	5
	C11	Environmental hygiene	0.145	3
	**C12**	**Embankment type**	**0.188**	**1**
	**C13**	**Water landscape aesthetics**	**0.148**	**1**
Mental health impact factor	**C14**	**Variety of recreational and fitness facilities**	**0.192**	**2**
	**C15**	**Number of recreational and fitness facilities**	**0.303**	**1**
	**C16**	**Number of scenic facilities**	**0.229**	**1**
	**C17**	**Quality of Scenic Facilities**	**0.251**	**1**
	C18	Activity area size	0.145	3
	**C19**	**Number of resting facilities**	**0.244**	**1**

*The bolded values in the table indicates that the factor’s score is significant.

Overall, Olympic Park ranked first among the seven parks in restorative effectiveness, with excellent performance. Indicators were well balanced, with social interaction health factors playing the dominant role, while psychological and physiological health factors provided strong support. The park demonstrated no significant weaknesses.

### 3.3 Optimization strategies for health restoration in waterfront parks along the Hunhe River, Shenyang

Based on the evaluation results of health restoration and the analysis of factor weights (Table 34), the studied parks can be classified into three categories.

(1) Parks dominated by psychological health restoration (Heping Sports Park, Hunnan Citizen Park). Their restorative function is primarily driven by perceptual factors, yet the lack of activity facilities constrains overall effectiveness. Optimization should focus on addressing these facility deficiencies and enhancing psychological experience through landscape and atmosphere design.
(2) Parks dominated by physiological health restoration (Luoshichuan Ecological Park, Changbai Island Forest Park). Here, natural ecological elements contribute most to restorative effects. Optimization should build upon existing ecological resources, further strengthening natural factors to enhance physiological well-being.
(3) Parks dominated by social interaction health restoration (Olympic Park, Shenshuiwan Park, Wulihe Park). These parks demonstrate well-balanced indicators with few weaknesses, and current conditions already support strong restorative effects. Optimization should emphasize design interventions and activity programming to attract more visitors and sustain park vitality.

**Table 34:** Scores of each factor of all parks.

			Luoshichuan Ecological Park	Shenshuiwan Park	Wulihe Park	Changbai Island Forest Park	Heping Sports Park	Hunnan Citizen Park	Olympic Park
C1	Mental health impact factor	Sense of spatial security	0.111	0.148	0.157	0.145	0.143	0.131	**0.152**
C2		Spatial privacy	0.113	0.165	0.151	0.135	0.137	0.122	**0.196**
C3		Openness of view	0.113	0.093	0.112	0.114	**0.202**	**0.188**	0.181
C4		Green view ratio	**0.161**	0.143	0.126	0.129	**0.155**	0.150	0.138
C5		Road connectivity	0.129	0.146	0.136	**0.148**	0.143	**0.148**	**0.151**
C6		Density of slow-moving road network	0.134	**0.169**	0.143	**0.144**	0.124	0.142	**0.144**
C7		Visual colorfulness	**0.181**	0.139	0.144	0.109	0.148	**0.167**	0.113
C8	Physiological health impact factor	Vegetation type	0.130	**0.217**	0.130	**0.217**	0.087	0.087	0.131
C9		Vegetation coverage rate	**0.163**	0.116	0.124	**0.177**	0.151	0.122	0.147
C10		Environmental quietness	**0.175**	0.123	0.136	0.127	**0.163**	0.141	0.133
C11		Environmental hygiene	**0.180**	0.132	0.116	0.134	**0.152**	0.141	0.145
C12		Embankment type	0.125	0.125	**0.188**	**0.188**	0.031	0.156	**0.188**
C13		Water landscape aesthetics	0.121	**0.148**	**0.148**	**0.148**	**0.148**	0.142	**0.148**
C14	Mental health impact factor	Variety of recreational and fitness facilities	0.038	**0.192**	**0.231**	0.154	0.038	0.154	**0.192**
C15		Number of recreational and fitness facilities	0.031	**0.273**	0.212	0.121	0.045	0.061	**0.303**
C16		Number of scenic facilities	0.105	**0.214**	**0.213**	0.116	0.121	0.051	**0.229**
C17		Quality of scenic facilities	0.077	**0.221**	0.150	0.112	0.152	0.054	**0.251**
C18		Activity area size	0.137	0.133	0.122	0.090	**0.232**	**0.204**	0.145
C19		Number of resting facilities	0.137	**0.205**	0.192	0.103	0.055	0.068	**0.244**

*The bolded values in the table indicates that the factor’s score and ranking are significant.

#### 3.3.1 Optimization strategies for parks dominated by psychological health restoration

Heping Sports Park and Hunnan Citizen Park ranked in the third tier of overall restorative effectiveness. Their restorative benefits are primarily derived from psychological health factors, but the lack of recreational and fitness facilities as well as limited activity space restrict their potential. Optimization should focus on improving the system of fitness facilities and the structure of activity spaces, while also enhancing spatial clustering and visual engagement to strengthen perceptual experience and encourage frequent use.

##### 3.3.1.1 Optimization of fitness facilities and activity spaces

Given the low scores of recreational and fitness facilities, it is recommended to introduce a wider range of facilities to increase diversity and attractiveness of activities. Although existing activity spaces are of relatively good size and landscape quality, most are enclosed natural grassland areas lacking essential amenities such as lighting, seating, and shaded areas, thereby reducing safety and convenience. Additional interventions could include installing interactive fitness stations, creating walking/jogging loops, and integrating small-scale sheltered seating to enhance usability and comfort. Thus, in addition to new facilities, artificially designed activity spaces should be introduced to complement natural ones, forming a mixed layout that improves adaptability to different user groups and enhances the capacity to support restorative health behaviors. Attention to visual openness and greenery placement can further enhance the restorative atmosphere.

##### 3.3.1.2 Construction of core activity spaces

Single facilities or isolated activity areas have limited impact in triggering restorative activities, while multi-functional composite spaces can significantly increase usage through clustering effects. Core spaces should be composed of both recreational/fitness facilities and activity grounds, with moderate spatial separation to prevent interference among different activities. In terms of layout, priority should be given to locations where recreational facilities and activity spaces overlap or are adjacent. Drawing from the typical case of Shenshuiwan Park, suitability assessments and integrative design should be conducted to adapt to local conditions, including optimizing sightlines, circulation paths, and vegetation layout to enhance perceived safety and engagement, thereby maximizing the psychological restorative value of waterfront parks.

#### 3.3.2 Optimization strategies for parks dominated by physiological health restoration

Luoshichuan Ecological Park and Changbai Island Forest Park were ranked in the second tier of restorative effectiveness. Their restorative function is primarily driven by physiological health factors, with natural environments-particularly water bodies and vegetation landscapes-contributing most to restorative experiences. Optimization strategies should focus on reinforcing ecological resource advantages and enhancing the usability and accessibility of natural features to maximize physiological restoration.

##### 3.3.2.1 Waterfront shoreline optimization

Benefiting from the Hun River system, the waterfront parks enjoy excellent hydrophilic conditions. According to the weight analysis of indicators, revetment type and shoreline accessibility to water account for a significant share of the physiological health restoration factors. Optimization can include creating gently sloped or stepped access points to water, adding floating or semi-floating platforms for recreation, and designing shoreline curves to provide varied visual and physical experiences. Vegetation along the shoreline should be layered to enhance ecological quality while maintaining clear sightlines for safety and comfort. The continuity of the waterfront shoreline can connect a variety of water-edge activity scenarios, thereby creating a health restoration corridor that combines ecological qualities with experiential richness.

##### 3.3.2.2 Health-oriented park design

Taking Luoshichuan Ecological Park as an example, the concept of restorative landscapes may be introduced to integrate fitness facilities, activity grounds, and hydrophilic spaces, while linking them through a continuous pathway system. The internal spatial structure should primarily consist of dense tree stands and landscape belts, interspersed with sunlit lawns, thus forming a gradient of open, semi-private, and private spaces. Additional strategies could include placing small rest nodes and exercise stations along pathways, designing shaded seating areas near water views, and ensuring smooth circulation between forested and open areas to encourage movement and engagement with the environment. This spatial transition would enable visitors to sequentially experience lively social interaction, tranquil forest settings, and soothing waterscapes, facilitating both physical and mental relaxation. The aim is to create an urban waterfront park with therapeutic functions, making full use of ecological resources to provide a high-quality environment for physiological health restoration.

#### 3.3.3 Optimization strategies for parks dominated by social interaction health restoration

Shenshuiwan Park, Wulihe Park, and Olympic Park demonstrated balanced performance in restorative evaluation, with high scores across dimensions and a significant capacity to foster social interaction. Their optimization should therefore focus on enhancing park vitality and clustering effects, creating spaces where social engagement naturally translates into restorative experiences without relying solely on recreational facilities.

##### 3.3.3.1 Spatial and cultural programming for vitality enhancement

Drawing lessons from successful international waterfront districts, strategies should emphasize iconic architecture, cultural facilities, and public activity spaces to attract visitors and generate high popularity along the waterfront. Specific interventions can include creating multifunctional plazas and open-air performance spaces strategically positioned near pedestrian paths and scenic spots to encourage both planned and spontaneous social gatherings. In terms of functional planning, it is essential to align with the city’s cultural atmosphere and avoid substituting health-oriented functions with purely commercial uses. The introduction of thematic cultural pavilions, temporary exhibition areas, and interactive art installations can stimulate repeated visits and provide diverse social contexts, thereby reinforcing social engagement and perceptual stimulation.

##### 3.3.3.2 Sustaining vitality through diverse and health-oriented activities

The attraction of landmark cultural facilities tends to decline over time, requiring continuous input of diverse, personalized, and health-oriented activities to maintain popularity and restorative effects. Measures should be strategically integrated with the park’s existing landscape and recreational infrastructure: Adding facilities supporting physical activities, such as camping and picnic areas, specialized service facilities, and children’s play zones (e.g., splash pads, sand play areas) complemented by spaces meeting parents’ health needs; Establishing fishing platforms to form hobby-oriented clusters; Leveraging the river and shoreline for accessible water-based activities and small-scale event nodes that connect multiple parks sections, encouraging circulation and interaction. Distinctive events can include marathons, cycling races, dragon boat races, sailing competitions, and winter ice sports; incorporating trendy leisure activities such as camping, picnicking, and photography-themed events. Rotating or seasonal programming ensures continual novelty and visitor engagement. By designing activities that are periodic, innovative, and participatory, parks can continuously attract residents, thereby sustaining and reinforcing the health restoration effects of social interaction.

## 4 Discussion

### 4.1 Key research findings

Taking Attention Restoration Theory as the entry point, this study systematically analyzed the health-restorative characteristics of the riverside parks along the Hunhe River in Shenyang. By combining quantitative evaluation with type-specific optimization strategies, the following key findings were obtained:

(1) Through principal component analysis, the influencing factors of health restoration in riverside parks were categorized into three dimensions: psychological health restoration, physiological health restoration, and social interaction health restoration. Regression analysis was then used to determine the relative weights of these dimensions, showing that their influence follows the order: psychological health restoration > physiological health restoration > social interaction health restoration.
(2) Based on the restorative environmental features of the Hunhe riverside parks, an evaluation index system was established comprising 19 indicators. Correlation analysis revealed that seven indicators-C3 visual openness, C7 visual color perception, C13 water landscape attractiveness, C12 revetment type, C14 diversity of recreational and fitness facilities, C15 quantity of recreational and fitness facilities, and C18 activity space area-were significantly correlated with health restoration. However, one counterintuitive finding-the weak negative correlation between visual colorfulness and restorative perception-requires further interpretation. This phenomenon may result from a combination of environmental and seasonal factors. In this study, visual colorfulness was quantified by extracting the dominant hue from park photographs using the Toolsou tool, where higher hue values indicate cooler tones. Parks with higher hue values, such as Luoshiquan Ecological Park, typically exhibit extensive cool-toned vegetation and limited chromatic diversity. According to the Attention Restoration Theory, overly cool or monotonous color environments may diminish the “fascination” dimension of restorative experience, thereby reducing psychological restoration. Furthermore, the field survey was conducted between September and October, coinciding with leaf senescence in Shenyang, when vegetation color becomes heterogeneous and visually cluttered, leading to an overall cooler and dimmer palette. Such visual and seasonal cues tend to reduce activity duration, limit social interactions, and lower engagement, which may generate mild psychological resistance or discomfort and ultimately decrease perceived restoration. Therefore, the timing of data collection (early autumn) may have amplified this effect. Future studies should consider multi-seasonal surveys, experimental manipulation of color environments, and inclusion of physiological indicators to validate these mechanisms.
(3) A comprehensive evaluation of the seven representative riverside parks showed the following ranking: Olympic Park > Shenshuiwan Park > Wulihe Park > Changbai Island Forest Park > Luoshichuan Ecological Park > Hunnan Citizen Park > Heping Sports Park. Among them, the first tier includes Olympic Park, Shenshuiwan Park, and Wulihe Park; the second tier includes Changbai Island Forest Park and Luoshichuan Ecological Park; and the third tier includes Hunnan Citizen Park and Heping Sports Park.
(4) According to the dominant factor types of health restoration, the seven parks can be divided into three categories: Psychological health restoration-dominated parks (Heping Sports Park, Hunnan Citizen Park), which require improvements in recreational facilities and optimization of perceptual experiences; Physiological health restoration-dominated parks (Changbai Island Forest Park, Luoshichuan Ecological Park), which should strengthen natural environmental factors and ecological health-care functions; Social interaction health restoration-dominated parks (Olympic Park, Shenshuiwan Park, Wulihe Park), which should enhance vitality through activity programming and spatial design. The differentiation of restorative functions among the seven parks is closely associated with both Shenyang’s “One River, Two Banks” development strategy and the surrounding land-use context. This city-scale strategy positions the Hunhe River as a central ecological and development corridor, guiding the spatial organization of diverse functional zones and cultural landscapes along its banks. Within this framework, adjacent land-use composition plays a decisive role in shaping restorative orientation. Specifically, Shen Shui Park, Wulihe Park, and Olympic Park are located in the urban core, where highly mixed land uses and vibrant cultural–commercial activities foster frequent social interactions, resulting in a socially interactive–oriented profile. In contrast, Luoshiquan and Changbai Island Parks, situated in the western residential–ecological belt, provide tranquil environments with high vegetation coverage, supporting stronger physiological restoration. Meanwhile, Heping Sports Park and Hunnan Sports Park, both adjacent to large sports facilities, show activity patterns dominated by exercise and recreation, aligning more closely with psychological restoration. Although this interpretation is primarily based on qualitative spatial analysis rather than rigorous quantitative modeling, it highlights how city-scale planning strategies and local spatial contexts jointly shape the restorative experience within urban parks.
(5) Based on the above classification and indicator weight analysis, targeted optimization strategies were proposed: For psychological health restoration-dominated parks, the focus should be on supplementing facilities and improving sensory experiences; For physiological health restoration-dominated parks, the emphasis should be on shoreline optimization and strengthening health-care functions; For social interaction health restoration-dominated parks, the priority should be activity-driven attraction and enhancement of social spaces.

### 4.2 Limitations and future outlook

Building upon the key findings presented in “Section 4.1,” this study has identified differentiated restorative profiles among the seven riverside parks, revealing how park typologies, environmental features, and surrounding land-use contexts jointly influence psychological, physiological, and social interaction health restoration. While these insights offer valuable guidance for both theory and practice, it is important to acknowledge that several limitations remain, which warrant further investigation and reflection:

(1) Insufficient depth and breadth of data. Although the research scope is broad, due to limitations of time and resources, the number of distributed questionnaires was limited, and the depth of investigation in some parks was insufficient. The sample size of 64 valid questionnaires limits the generalizability of the findings to broader populations. While the survey focused on frequent users of each park to ensure representative sampling and enhance the precision of behavioral perception data, this small-sample precise approach may still not capture the full diversity of visitor experiences. Future research could expand the sample size, adopt stratified sampling across different user groups, and combine long-term behavioral observation or physiological measurements to further validate and generalize the restorative evaluation framework. In addition, certain case data relied on online sources, which may lead to discrepancies between the results and actual conditions.
(2) Incomplete consideration of the complexity of human health. Human health is influenced by multiple factors, including physical fitness, genetic background, and lifestyle habits. While the natural environment can facilitate health restoration, its effects are limited. For feasibility reasons, this study did not incorporate such individual differences into the model. Future research may integrate multi-dimensional health data to enable more comprehensive analyses. In addition, this study did not fully account for the influence of temporal and climatic variations. Seasonal changes in temperature, lighting, and landscape color can significantly affect spatial visual richness and residents’ behavioral patterns. During the transitional period from late summer to autumn, for instance, cooler temperatures may alter.
(3) Travel modes and activity frequencies, while people’s psychological perceptions, social interactions, and physiological needs also shift accordingly. Future research should therefore incorporate cross-seasonal and temporal comparisons to reveal the dynamic relationships between visual environments and human behaviors more comprehensively.
(4) Single evaluation method for health restoration. Due to site constraints, this study primarily relied on subjective scale-based assessments, lacking objective monitoring tools such as electrocardiogram sensors. As a result, the evaluation outcomes may be affected by respondents’ interpretations and perceptual biases. Future studies could adopt a combined subjective–objective evaluation approach, incorporating physiological monitoring data to improve scientific rigor and accuracy.

## 5 Conclusion

This study develops a comprehensive evaluation framework that integrates objective environmental indicators with subjective perception data, providing a systematic approach to identifying the restorative environmental characteristics of urban waterfront parks. Empirical findings indicate that the key restorative environmental factors include spatial accessibility, ecological integrity, facility adequacy, and landscape perception, with natural elements exerting the most significant influence on residents’ restorative experiences. Behavioral surveys reveal that residents engage in diverse restorative activities, primarily leisure walking, quiet relaxation, and social interaction. The spatial distribution of these activities is strongly associated with the park’s pedestrian network, vegetation coverage, and water features.

Subjective perception assessments complement the limitations of objective indicators, highlighting public sensitivity to safety, color perception, and spatial openness, all of which significantly shape restorative experiences. In particular, the richness and layering of visual landscapes were found to enhance psychological restoration, underscoring the importance of multisensory design in waterfront environments. However, the visual and behavioral effects identified in this study may vary under different temporal and climatic conditions, suggesting that future research should incorporate seasonal and temporal dynamics to better understand the evolving relationship between environmental perception and human well-being.

The findings not only extend the theoretical application of restorative environments in the context of waterfront parks but also provide empirical evidence to guide the renewal and optimization of urban waterfront spaces, offering practical implications for policy and planning. Overall, this research contributes to the growing interdisciplinary dialogue between environmental psychology and urban design, emphasizing the need for more resilient, adaptive, and human-centered public spaces.

## Data Availability

The original contributions presented in the study are included in the article/supplementary material, further inquiries can be directed to the corresponding author.
